# SMA CARNI-VAL Trial Part I: Double-Blind, Randomized, Placebo-Controlled Trial of L-Carnitine and Valproic Acid in Spinal Muscular Atrophy

**DOI:** 10.1371/journal.pone.0012140

**Published:** 2010-08-19

**Authors:** Kathryn J. Swoboda, Charles B. Scott, Thomas O. Crawford, Louise R. Simard, Sandra P. Reyna, Kristin J. Krosschell, Gyula Acsadi, Bakri Elsheik, Mary K. Schroth, Guy D'Anjou, Bernard LaSalle, Thomas W. Prior, Susan L. Sorenson, Jo Anne Maczulski, Mark B. Bromberg, Gary M. Chan, John T. Kissel, for the Project Cure Spinal Muscular Atrophy Investigators Network

**Affiliations:** 1 Department of Neurology, University of Utah School of Medicine, Salt Lake City, Utah, United States of America; 2 CBS Squared, Inc, Fort Washington, Pennsylvania, United States of America; 3 Department of Biochemistry and Medical Genetics, University of Manitoba, Winnipeg, Manitoba, Canada; 4 Departments of Neurology and Pediatrics, Johns Hopkins University School of Medicine, Baltimore, Maryland, United States of America; 5 General Clinical Research Center, University of Utah School of Medicine, Salt Lake City, Utah, United States of America; 6 Department of Molecular Pathology, Ohio State University, Columbus, Ohio, United States of America; 7 Primary Children's Medical Center, Salt Lake City, Utah, United States of America; 8 Pediatric Occupational Therapy Services, Chicago, Illinois, United States of America; 9 Departments of Neurology and Pediatrics, Wayne State University School of Medicine, Detroit, Michigan, United States of America; 10 Department of Physical Therapy and Human Movement Sciences, Feinberg School of Medicine, Northwestern University, Chicago, Illinois, United States of America; 11 Division of Pediatric Neurology, Ste-Justine Hospital, Montréal, Québec, Canada; 12 Department of Pediatrics, University of Wisconsin School of Medicine and Public Health, Madison, Wisconsin, United States of America; 13 Department of Pediatric Neonatology, University of Utah, Salt Lake City, Utah, United States of America; 14 Department of Neurology, Ohio State University Medical Center, Columbus, Ohio, United States of America; University Paris Descartes, France

## Abstract

**Background:**

Valproic acid (VPA) has demonstrated potential as a therapeutic candidate for spinal muscular atrophy (SMA) *in vitro* and *in vivo*.

**Methods:**

Two cohorts of subjects were enrolled in the SMA CARNIVAL TRIAL, a non-ambulatory group of “sitters” (cohort 1) and an ambulatory group of “walkers” (cohort 2). Here, we present results for cohort 1: a multicenter phase II randomized double-blind intention-to-treat protocol in non-ambulatory SMA subjects 2–8 years of age. Sixty-one subjects were randomized 1∶1 to placebo or treatment for the first six months; all received active treatment the subsequent six months. The primary outcome was change in the modified Hammersmith Functional Motor Scale (MHFMS) score following six months of treatment. Secondary outcomes included safety and adverse event data, and change in MHFMS score for twelve versus six months of active treatment, body composition, quantitative SMN mRNA levels, maximum ulnar CMAP amplitudes, myometry and PFT measures.

**Results:**

At 6 months, there was no difference in change from the baseline MHFMS score between treatment and placebo groups (difference = 0.643, 95% CI = −1.22–2.51). Adverse events occurred in >80% of subjects and were more common in the treatment group. Excessive weight gain was the most frequent drug-related adverse event, and increased fat mass was negatively related to change in MHFMS values (p = 0.0409). Post-hoc analysis found that children ages two to three years that received 12 months treatment, when adjusted for baseline weight, had significantly improved MHFMS scores (p = 0.03) compared to those who received placebo the first six months. A linear regression analysis limited to the influence of age demonstrates young age as a significant factor in improved MHFMS scores (p = 0.007).

**Conclusions:**

This study demonstrated no benefit from six months treatment with VPA and L-carnitine in a young non-ambulatory cohort of subjects with SMA. Weight gain, age and treatment duration were significant confounding variables that should be considered in the design of future trials.

**Trial Registry:**

Clinicaltrials.gov NCT00227266

## Introduction

Spinal muscular atrophy (SMA) is an autosomal recessive motor neuron disease and a leading cause of infant and childhood mortality. [1]–[6] More than 95% of affected individuals demonstrate a homozygous deletion/mutation involving exon 7 in *SMN1* (survival motor neuron 1), resulting in the biochemical deficiency of the SMN protein, part of a complex that functions in the assembly of small nuclear ribonucleoprotein particles (snRNP). [7], [8] A genomic duplication at this locus has produced a nearly identical gene, *SMN2* (survival motor neuron 2) that differs from *SMN1* by a nucleotide substitution that promotes exon 7 exclusion, thus producing only a fraction of the identical full length protein. Phenotypic variation in SMA correlates with the number of *SMN2* gene copies and the level of SMN protein in cells [9]–[15].

Because all SMA patients have 1 or more *SMN2* gene copies, small molecule compounds that target *SMN2* to produce increased quantities of full-length SMN protein from the existing *SMN2* gene(s) are attractive therapeutic candidates. Valproic acid (VPA), a histone deacetylase (HDAC) inhibitor, directly increases SMN expression in SMA patient-derived cell lines in vitro. [16], [17] Preliminary *in vivo* data in humans demonstrates up-regulation of *SMN2* expression in about one-third of SMA subjects via inhibition of HDAC2 and also appears to alter splicing to increase SMN7 inclusion and thus full-length SMN protein. [18], [19] VPA has also been demonstrated to have neuroprotective properties on glutamate-induced excitotoxicity via up-regulation of alpha-synuclein and increases neurite outgrowth in vitro. [20], [21] VPA treatment has been demonstrated to increase survival in animal models of motor neuron disease including amyotrophic lateral sclerosis and SMA. [22]–[24] In an SMA mouse model, VPA treatment resulted in improved gross motor function, larger evoked motor potentials, less degeneration of spinal motor neurons and improved neuromuscular junction innervation in treated animals compared to age-matched controls [24]. Three open label trials of VPA in human subjects have been published to date, all indicating a possible modest benefit in strength and/or motor function [25]–[27].

As children with SMA may have a limited carnitine synthetic capacity due to significantly diminished skeletal muscle mass, and VPA is known to inhibit carnitine transport and deplete carnitine levels by binding to VPA metabolites, we elected to combine VPA therapy with sufficient supplemental carnitine to avoid concerns about a confounding effect of carnitine depletion. This decision was influenced by data from our open label VPA trial, which indicated an increased suspectibility in SMA subjects to carnitine depletion with VPA treatment [27].

Our primary objective was to assess potential benefit for improving motor function in a young non-ambulatory cohort of children with SMA in a randomized double-blind placebo-controlled clinical trial. Additional objectives were to further assess the safety of VPA in children with SMA, to assess performance of selected outcome measures for use in a multi-center clinical trials setting and to look for evidence that would support a biologic effect of VPA in these subjects, such as changes in SMN expression, electrophysiologic outcomes, or both.

## Methods

### Ethics Statement

Written informed parental consent (subjects <18 years) and assent (subjects ≥7 years) were obtained for all subjects. The study was approved by the University of Utah Institutional Review Board (IRB) and at each participating clinical trial site (University of Wisconsin-Madison Health Sciences; Wayne State University; Ohio State Biomedical; Johns Hopkins Medical and Ste Justine Hospital.

### Trial Design

The SMA CARNI-VAL trial was a multi-center phase II trial of CARNItine and VALproic acid in patients with spinal muscular atrophy. This trial consisted of two parallel multi-center studies, targeting different SMA cohorts (Clinicaltrials.gov ID NCT00227266). The protocol for this trial and supporting CONSORT checklist are available as supporting information; see Checklist S1 and Protocol S1. Part 1 was a prospective double blind, placebo-controlled randomized intention-to-treat protocol to assess safety and efficacy of VPA and L-carnitine in non-ambulatory SMA type II or III “sitters” 2–8 years of age (cohort 1). Part 2 was a parallel open label trial in SMA type II or III “standers and walkers” 3–17 years of age (cohort 2). In this paper, we review objectives, methods and results only for Part 1.

### Study population

**Figure 1:**
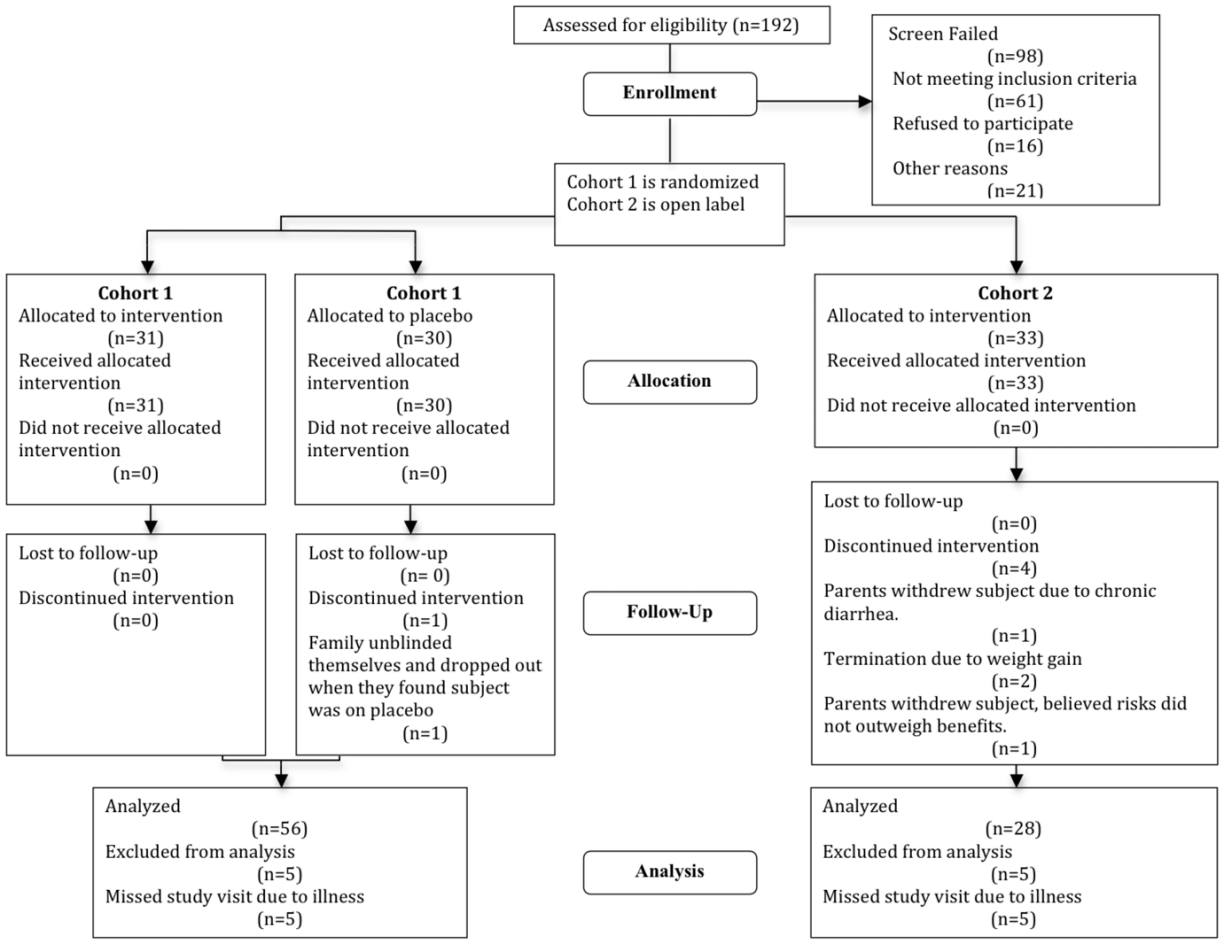
Consort flow diagram.

**Figure 2:**
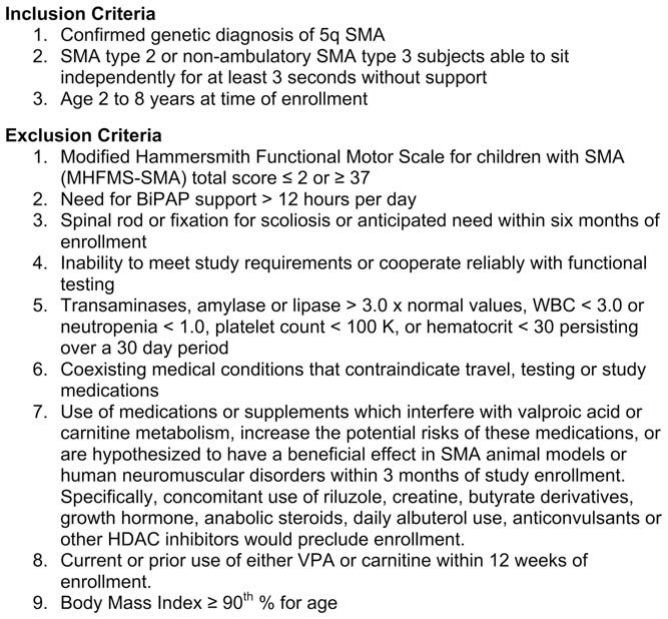
Inclusion and exclusion criteria.

We prospectively enrolled 61 non-ambulatory SMA children at six centers in North America. The progress of all participants through the trial is diagrammed in Figure 1. Inclusion and exclusion criteria are indicated in Figure 2.

### Study procedures

All subjects completed two baseline visits within a two to six week period. Following the second visit, subjects were randomized to either the placebo arm (group 1) or the treatment arm (group 2). There were 31 subjects randomized to the placebo arm and 30 subjects randomized to the active treatment arm (Figure 1). Placebo was provided for both L-carnitine and VPA. Subjects and investigators remained blinded for the duration of the study. At the six-month visit, subjects in group 1 were switched to active treatment for the second six-month period, and those in group 2 continued on study medication for an additional 6 months. Thus, half of the children received treatment for the last 6 months of the study, and half received active treatment for the full 12 months. This trial design was intended to improve trial enrollment and compliance with the protocol, as focus group discussions with parents of eligible children indicated significant reluctance to enroll in a trial of a licensed drug in which there was a 50% chance of receiving only placebo. Nonetheless, extension of the trial to 12 months in this manner was thought to be potentially useful to study both the benefit, and burden, of longer treatment duration.

Abbott Pharmaceutical provided VPA and placebo, and Sigma-Tau Pharmaceutical provided L-carnitine, at no cost. Divalproex sodium coated particles (Depakote® sprinkle capsules, 125 mg) or matched placebo was administered in divided doses two to three times daily sufficient to maintain overnight trough levels of 50–100 mg/dL. L-carnitine (or matched placebo, 100 mg/ml liquid) was dosed at 50 mg/kg/day, to a maximum of 1000 mg, divided into two daily doses.

Treatment assessments were performed at 3 (V1), 6 (V2) and 12 (V3) months. Laboratories were performed at baseline, 2–3 weeks following initiation, at each treatment visit and midway between V2 and V3 visits, and included a basic chemistry profile, CBC with platelets, transaminases, carnitine profile, amylase, lipase and trough VPA levels. An un-blinded medical monitor, who also reviewed subjects' blood tests and adverse events, performed dosing adjustments. Similar adjustments were made for subjects in the placebo group to preserve blinded status of participants. Contact was possible between the unblinded medical monitor and patients if required by clinical concerns, but efforts were taken to avoid unblinding of the site investigator, parents and patient.

### Outcome measures

Primary outcome measures included laboratory safety data, adverse event data and efficacy as measured by change from baseline Modified Hammersmith Functional Motor Scale (MHFMS) scores following six months of treatment versus placebo. Secondary outcome measures included change from baseline MHFMS score following 12 versus 6 months treatment, estimates of innervation via maximum ulnar compound muscle action potential (CMAP), dual-energy X-ray absorptiometry (DEXA), evaluation of body composition and bone density, quantitative assessment of SMN mRNA; evaluation of quality of life using the Pediatric Quality of Life Inventory (PedsQL) and for children five years and older, change from baseline measures of pulmonary function and muscle strength via handheld myometry at 6 and 12 months.

Gross motor function was assessed with the MHFMS. This scale was previously modified for use in a research setting but contains the same 20 gross motor function items as the original clinical scale (complete protocol available at http://smaoutcomes.org). [28]–[29] Degree of innervation by the ulnar nerve in the hypothenar muscle group of the hand was estimated using maximum ulnar CMAP amplitude (complete CMAP protocol available at http://smaoutcomes.org). In children five years and older, myometry measurements were performed three times for right and left elbow flexion, and for right and left knee extension, at each visit using the Lafayette Instrument MMT System Model 01163 myometer with a positioning method as previously described. [30], [31] If any measure was more than 10% different than any of the other two measurements, then a fourth measurement was performed. The summary statistic was the average of either three or four measurements taken during a visit for both the right and left elbow and knee. [32]–[34] In children five years and older, pulmonary function testing (PFT) was performed in an accredited pediatric laboratory and included forced vital capacity (FVC), forced expiratory volume in one second (FEV1) and maximum expiratory and inspiratory pressures (MEP, MIP). Dual-energy X-ray absorptiometry (DEXA) scanning for bone density and body composition was performed at the Salt Lake City, Madison and Columbus sites. Norland DEXA XR-36 software version 3.3.1 for small subjects was used to assess whole body composition and bone mineral density (BMD) and bone mineral content (BMC). Quality of life (QOL) was assessed using the PedsQL [35]. The same parent completed the PedsQL at each visit and children five years of age and older completed the age-appropriate PedsQL. The questionnaire is scored on a 0 to 100 scale such that a higher score indicates better QOL.

*SMN2* copy number was determined as previously described. [11] Whole blood was drawn into PAXgene tubes at baseline and each visit and quantitation of full length SMN (flSMN) and SMNΔ7 mRNA transcripts was performed as previously described. [27], [36] Human RPLPO (large ribosomal protein) and PGK1 (phosphoglycerate kinase 1) were run as endogenous controls. Subjects missing baseline data or who had incomplete visit data were excluded from this analysis. Results are reported as relative amounts of flSMN or SMNΔ7 transcripts normalized against the relative amount of RPLPO.

### Adverse events data management

Adverse events were elicited systematically via a full review of systems at the time of each visit, and parents were instructed to immediately report symptoms or illnesses occurring between visits via telephone. Study coordinators also reviewed medical records for emergency room or hospital visits. Adverse events were graded using Common Terminology Criteria for Adverse Events v3.0 (CTCAE v3.0). An independent Data and Safety Monitoring Committee provided oversight for the study.

### Statistical Analysis

A prior open-label study of SMA type 2 subjects showed the standard deviation for MHFMS-SMA was 3.19. [27] A clinically meaningful change was estimated to be a 3 point change in MHFMS-SMA at 6 months. A sample size of 25 subjects completing both the baseline and six-month evaluations provided at least 90% statistical power with an effect size of 0.941, Type I error of 0.05 (two-sided). Assuming 20% attrition then 60 total subjects were required. Subjects were randomized using a permuted block design with blocks of size 6 with no stratification. The statistician for the study (author CS) created a blinded randomization list using the permuted block algorithm. At the second screening visit the site determines whether the subject was eligible, then completed a randomization request form that was faxed to one of the authors (CS) who would assign the subject per the predetermined randomization list. The treatment assignment was then sent to the central pharmacy to dispense the study medication. The statistician was blinded to treatment assignment, as was the study coordinator, treating investigator, patient and parents.

The assumption of normality for continuous variables was examined using the Shapiro-Wilks tests. Fisher's exact test was used to compare treatment groups in the analysis of categorical variables (e.g., gender). Baseline demographics and function were analyzed as continuous variables with treatment groups compared using a t-test. Analysis of variance (ANOVA) test was used to compare treatment groups for change from baseline data when normally distributed (e.g., change in MHFMS at 6 months). If the data were not normally distributed then the Wilcoxon rank-sum test was used (e.g., change in BMD). Linear regression analysis was used to examine baseline characteristics, potential prognostic factors for normally distributed outcome variables. The Pearson Correlation Coefficient, calculated from the linear regression, was used to assess association between continuous variables. Longitudinal analysis was performed using generalized estimating equations. When the data were normally distributed the identity link function was used and unstructured correlation. We used the small sample adjustment to the Score test, J/(J-1) where J is the number of clusters [37] When both random and fixed effects were analyzed, a mixed effect longitudinal analysis was performed. A p-value less than 0.05 was considered statistically significant. Reliability of the MHFMS in this group of subjects was evaluated using the intraclass correlation coefficient (ICC). This was used to determine the reliability across evaluators at different sites.

All analyses were performed on an intent-to-treat population that was defined as all subjects randomized to receive study medication. There were 61 subjects randomized, 4 subjects did not have a 6-month data: 2 of these dropped out after the 3-month visit, the other 2 missed their 6-month visit due to illness. At 12 months the 2 subjects missing the 6 month visit returned. MHFMS was not performed in one additional patient at 6 months. At 12-months one had a missing MHFMS. No data imputation was performed. CMAP was not performed according to protocol at 2 of the clinical sites accounting for 17 missing values. Parent-proxy QOL was not collected at the 6 month visit in 3 subjects.

## Results

Recruitment began in September 2005 and enrollment of the last subject was completed in October of 2006. The last subjects' final visit was in November 2007, and database was locked in March 2008. Participant flow is outlined in figure 1.

### Baseline Characteristics of Study Population

The analysis of demographic data (Table 1) indicates that the 2 treatments were well balanced for most factors. More males were enrolled in the placebo group (64.5%) and more females were enrolled in the treatment group (56.7%) but overall, there was no statistical difference (p = , 0.126). Body Mass Index (BMI) assessments indicated no significant difference between treatment arms (Supplemental Table S1). Total body BMD, BMC, lean mass and fat mass prior to the start of treatment were assessed via DEXA scans performed at three sites (Supplemental Table S2). Baseline pulmonary function tests (PFTs) in subjects five years of age and older and baseline CMAP values were equivalent across groups (Supplemental Tables S3 and S4). Myometry data from children five years of age and older indicated that upper extremity strength was greater in the placebo group (Supplemental Table S5, p≤.03), but lower extremity myometry data demonstrated no difference between groups. The PedsQL parent assessment indicates no statistical difference between the placebo and treatment group on any of the subscales, psychosocial or total QOL (Supplemental Table S6). Only a subset of subjects was able to complete their own PedsQL (Supplemental Table S7).

**Table 1:** Baseline demographics by treatment arm.

	Group 1 Placebo^1^		Group 2 CARNI-VAL^2^		Total	
Characteristic	N = 31	(%)	N = 30	(%)	N = 61	(%)
Age (years)						
Mean	**4.4**		**4.3**		**4.3**	
SD	1.9		2.1		2.0	
Median	4.1		3.7		3.8	
Range	2.1–7.9		1.8–8.7		1.8–8.7	
Gender						
Female	11	(35.5)	17	(56.7)	28	(45.9)
Male	20	(64.5)	13	(43.3)	33	(54.1)
Ethnicity						
Hispanic	2	(6.5)	1	(3.3)	3	(4.9)
Non-Hispanic	29	(93.5)	27	(90.0)	56	(91.8)
Unknown	0	(0.0)	2	(6.7)	2	(3.3)
Race						
Asian	1	(3.2)	2	(6.7)	3	(4.9)
African American	1	(3.2)	0	(0.0)	1	(1.6)
White	26	(83.9)	25	(83.3)	51	(83.6)
Unknown	3	(9.7)	3	(10.0)	6	(9.8)

1 =  placebo group received matched placebo for both medications, L-carnitine and VPA.

2 = active treatment group received both L-carnitine and VPA.

### Reliability of the primary efficacy outcome measure, MHFMS-SMA, during the screening period

We used the ICC to determine the reliability of MHFMS from the first screening visit to the second, rated within their institution. Evaluating all subjects, the reliability is 0.97. The reliability at each institution was excellent and similar to the reliability previously reported (0.95–0.99). [27], [29]

### Primary efficacy outcome

#### Impact of treatment on gross motor function as measured via MHFMS

The primary endpoint was change from baseline in the MHFMS score at 6 months. The distribution of the change from baseline data was normally distributed. There was no difference in change from baseline at 6 months between placebo and treatment groups (p = 0.492, Table 2). A linear regression analysis for the change from baseline to 6 months of the MHFMS, including age at study entry and treatment group as independent variables, indicates that age has a borderline effect on change (p = 0.0564), but treatment was not significant at p = 0.0912. The coefficient for age is -0.47 (SE 0.238) indicating that as age increases the change in MHFMS decreases. Thus, subjects that were older at the start of the study had less of an increase in scores than younger subjects. Gender was examined as a possible independent variable but was excluded from the model (p = 0.92).

**Table 2:** MHFMS score – Change from baseline to 6 months (Phase I).

	Group 1 Placebo^1^	Group 2 CARNI-VAL^2^	
Endpoint	N = 31	N = 30	p-value
Last Screen Value (S2)			
Mean	**20.0**	**16.6**	0.1373
SD	9.3	8.7	
Median	18.0	16.5	
Range	3.0–38.0	3.0–36.0	
6 Month Visit (V2)			
Mean	**20.6**	**16.8**	0.0790
SD	8.1	7.9	
Median	21.0	16.0	
Range	5.0–36.0	4.0–33.0	
N	28	28	
Change From Baseline			
Mean*	**0.18**	**0.82**	0.4921
SD	3.98	2.88	
Median	0.00	1.00	
Range	−12.0–9.0	−7.0–7.0	

1 =  placebo group received matched placebo for both medications, L-carnitine and VPA.

2 = active treatment group received both L-carnitine and VPA.

MHFMS = Modified Hammersmith Functional Motor Scale Score (Range 0–40).

*Difference  = 0.643: Upper confidence level difference 2.505, Lower confidence level difference −1.219.

During the last six months, starting from V2, all subjects received active treatment. There was no statistical difference in the change from baseline between the two groups; however, for subjects on treatment for the entire 12 months, there was a small increase in the MHFMS score compared to baseline (Table 3, p = 0.185). Based upon the indication that age had a negative relationship on MHFMS change over time, we investigated splitting the subjects into two groups: over age three years and under age three years. Generalized estimating equations were used to examine the change from baseline MHFMS score over a 12 month period in children ages two to three years of age (n = 23). There was a negative effect of baseline weight over the 12 month interval (estimate = −0.81, p = 0.0066) but a positive effect of treatment over that period (estimate = 1.21, p = 0.03). Analysis of children three years of age and older (n = 33) demonstrated a similar negative weight effect (estimate = −0.16, p = 0.0005) without the apparent treatment effect (p = 0.153). Table 4 displays the change from baseline in MHFMS within each age group by treatment arm. Subjects two to three years of age in the treatment group have an increasing slope through each visit, indicating a treatment benefit, while no such effect was observed in children greater than 3 years.

**Table 3:** MHFMS - Change from baseline to 12 months (Phases I and II).

	Group 1 Placebo Phase I* CARNI-VAL Phase II**	Group 2 CARNI-VAL Phase I* CARNI-VAL Phase II**	
Endpoint	N = 28	N = 30	p-value
Last Screen Value (S2)			
Mean	**20.0**	**16.6**	
SD	9.6	8.7	
Median	17.5	16.5	
Range	3.0–38.0	3.0–36.0	
12 Month Visit (V3)			
Mean	**20.1**	**18.3**	0.4039
SD	8.4	7.9	
Median	19.5	18.0	
Range	5.0–33.0	5.0–34.0	
Change From Baseline			
Mean***	**0.14**	**1.73**	0.1845
SD	4.68	4.31	
Median	0.00	2.00	
Range	−14.0–13.0	−8.0–12.0	

*Phase I  =  first six months treatment period in which subjects were randomized to receive either placebo for both VPA and L-carnitine or active treatment.

**Phase II  =  intention to treat period in which all subjects receive active treatment

MHFMS = Modified Hammersmith Functional Motor Scale Score (Range 0–40).

***Difference = 1.591. Upper confidence level difference 3.962, Lower confidence level difference −0.782.

**Table 4:** MHFMS within age groups by treatment arm – change from baseline.

	<3 years		3–8 years	
Change from Baseline Visit	Placebo^1^	CARNI-VAL^2^	Placebo^1^	CARNI-VAL^2^
V1 (3 month visit)	Phase I		Phase I	
N	11	10	20	18
Mean	**1.18**	**0.90**	**0.45**	**1.06**
SD	3.49	1.66	2.70	2.87
V2 (6 month visit)	Phase I		Phase I	
N	11	12	17	18
Mean	**1.09**	**1.33**	**−0.41**	**0.44**
SD	5.37	2.27	2.79	3.29
V3 (12 month visit)	Phase II		Phase II	
N	11	12	17	18
Mean	**2.09**	**2.92**	**−1.11**	**0.94**
SD	6.41	3.50	2.64	4.70

1 =  placebo group received matched placebo for both medications, L-carnitine and VPA for the first six months (Phase I), followed by treatment with L-carnitine and VPA the second six months (Phase II).

2 = active treatment group received both L-carnitine and VPA for the full twelve months.

MHFMS = Modified Hammersmith Functional Motor Scale Score.

### Ancillary analyses

#### Impact of weight, BMI and body composition on primary motor outcomes

**Figure 3:**
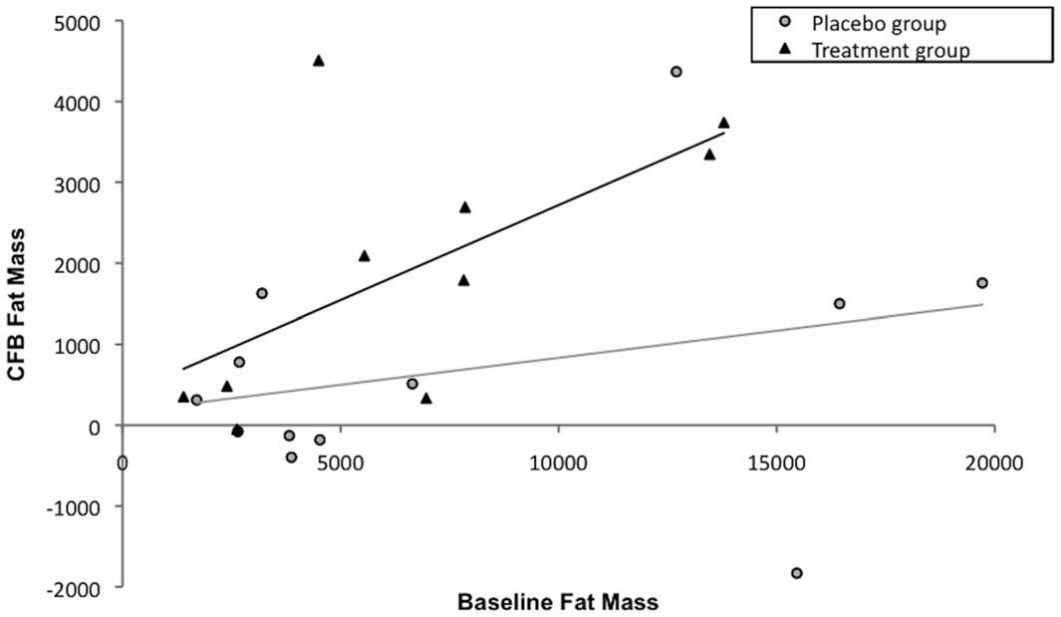
Change from baseline fat mass versus the baseline fat mass by treatment arm.

The BMI z-score and BMI percent were adjusted for subject age. BMI, BMI z-score and BMI percent were available at each follow-up visit. A generalized estimating equation analysis of change from baseline MHFMS indicated that increasing BMI over the year of study was negatively associated with outcome. BMI was treated as a time-varying covariate in the generalized estimating equation model. As BMI increased over time, change in MHFMS decreased (estimate = −0.05, p<0.0001). Linear regression analysis indicated that a change in fat mass as measured by DEXA is negatively related to a change in MHFMS (p = 0.0409). As fat mass increases by the 6-month time-point, MHFMS decreases. Figure 3 presents the change from baseline fat mass versus the baseline fat mass by treatment arm from the three sites that obtained DEXA measurements. BMI percent based upon z-score correlated well with baseline fat mass (correlation = 0.475, p = 0.0004). A regression analysis indicates that as weight at baseline increases, change from baseline MHFMS score worsens (p = 0.0058).

#### Impact of treatment on bone density

The change in total body BMD and BMC at 6 months was not normally distributed. There were no differences between the two treatment groups in the change in BMD (p = 0.82) or BMC (p = 0.41).

#### Impact of age on primary motor outcome

As subjects get older, their weight increases, and the relationship between weight and age causes a multi-colinearity problem in the analysis. However, linear regression analysis of change in MHFMS and age as the only independent predictor indicated that age is statistically significant at p = 0.0069. The coefficient for age is −0.62 (SE 0.219) indicating that as age increases the change in MHFMS score decreases.

#### Impact of treatment on electrophysiologic measures of innervation

**Figure 4:**
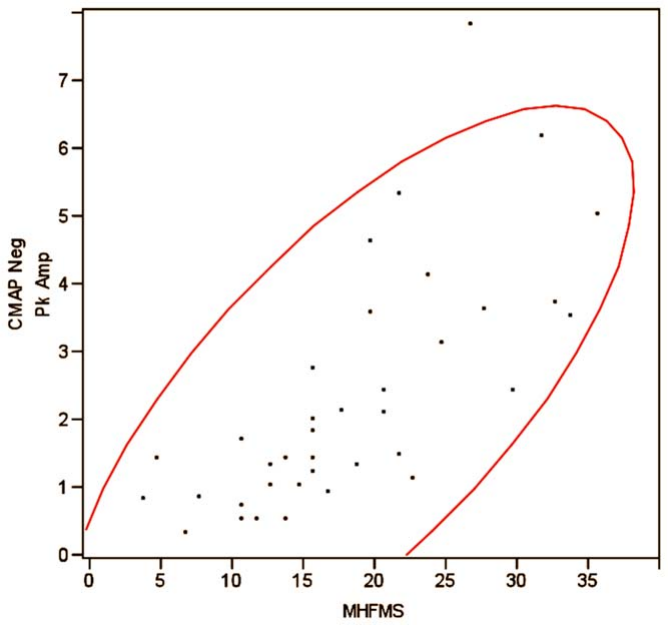
Scatterplot matrix demonstrating correlation between maximum CMAP negative peak amplitude and MHFMS scores with 95% density ellipse. Correlation  = 0.5643. The red lines are 95% confidence bands. Note that the majority of the data are contained in the linear relationship between these two factors with only one outlier.

There was no significant change in CMAP negative peak amplitude or area measurements between treatment groups after 6 months (Table 5). However, there was a good correlation between maximum CMAP amplitude and our primary outcome measure, the MHFMS (figure 4).

**Table 5:** CMAP values by treatment arm – 6 month values and change from baseline.

Characteristic	Placebo^1^ N = 31	CARNIVAL^2^ N = 30	Total N = 61
Compound Muscle Action Potential Amplitude at 6 Months (CMAP, mV)			
N	19	19	38
Mean	**2.32**	**2.37**	**2.34**
SD	1.75	1.82	1.76
Median	1.44	1.80	1.73
Range	0.50–6.14	0.30–7.81	0.30–7.81
CMAP Amplitude Change from Baseline (mV)			
N	19	19	38
Mean*	**−0.10**	**0.02**	**−0.04**
SD	0.66	0.70	0.67
Median	0.06	0.20	0.08
Range	−1.52–1.20	−1.70–1.15	−1.70–1.20
p-value (P vs C)	0.589		
p-value (Change from Zero)			0.715
Compound Muscle Action Potential Area at 6 Months (CMAP, mVus)			
N	19	19	38
Mean**	**5.28**	**5.26**	**5.27**
SD	4.49	4.65	4.51
Median	3.74	3.40	3.52
CMAP Area Change from Baseline (mVus)			
N	19	19	38
Mean	**−0.64**	**−0.07**	**−0.36**
SD	1.57	1.11	1.37
Median	−0.35	0.10	−0.15
Range	−3.60–2.09	−2.40–2.04	−3.60–2.09
p-value (P vs.C)	0.2046		
p-value (Change from Zero)			0.1138

1 =  placebo group received matched placebo for both medications, L-carnitine and VPA.

2 = active treatment group received both L-carnitine and VPA.

*Difference  = 0.121. Upper confidence level difference 0.568. Lower confidence level difference −0.328.

**Difference  = 0.570. Upper confidence level difference 1.465. Lower confidence level difference −0.326.

#### Impact of treatment on quality of life outcome assessments

Quality of life was measured using the PedsQL. There were no statistically significant differences between treatment groups for the parent-proxy QOL at 6 months (Table 6). The parent-proxy total QOL was compared to significant change (3 point change) in MHFMS at 6 months. Total QOL did not improve as MHFMS improved, but there was evidence of deterioration in QOL as MHFMS declined (Table 7).

**Table 6:** Parent-proxy assessment of quality of life (PedsQL) by treatment arm −6 month values and change from baseline.

	Placebo^1^	CARNIVAL^2^	Total
Characteristic	N = 31	N = 30	N = 61
School Functioning 6 Month Value			
N	23	22	45
Mean	**64.6**	**59.8**	**62.3**
SD	16.8	16.0	16.4
Median	60	60	60
Range	16.7–100	0–80	0–100
School Functioning Change from Baseline			
N	19	21	40
Mean	**−2.1**	**−8.9**	**−5.7**
SD	22.7	13.8	18.6
Median	0	−5	−5
Range	−50–35	−50–8.3	−50–35
Psychosocial Summary 6 Month Value			
N	27	27	54
Mean	**68.2**	**66.1**	**67.2**
SD	12.2	12.0	12.0
Median	66.7	68.2	67.4
Range	43.2–88.6	27.3–81.2	27.3–88.6
Psychosocial Summary Change from Baseline			
N	27	27	54
Mean	**0.2**	**2.5**	**−1.2**
SD	14.4	13.0	13.7
Median	0.8	0	0.4
Range	−31.6–31.8	−50–15.6	−50–31.8
Total QOL 6 Month Value			
N	27	27	54
Mean	**55.0**	**50.6**	**52.8**
SD	14.5	10.2	12.6
Median	51.2	51.7	51.5
Range	29.2–85	22.2–73.1	22.2–85
Total QOL Change from Baseline			
N	27	27	54
Mean	**0.3**	**−1.9**	**−0.8**
SD	12.9	13.6	13.1
Median	3.6	−1.2	0.8
Range	−32.2–31.9	−33.1–18.1	−33.1–31.9

1 =  placebo group received matched placebo for both medications, L-carnitine and VPA.

2 = active treatment group received both L-carnitine and VPA.

QOL = Quality of Life.

**Table 7:** MHFMS 3-point response and average parent-proxy total QOL - change from baseline to 6 months.

Level	Number	Mean	SD
Decline	7	−3.4127	10.2912
Stable	34	−0.7391	14.7807
Response	13	0.2991	10.1477

MHFMS = Modified Hammersmith Functional Motor Scale Score.

#### Impact of treatment on myometry measurements

Myometry was assessed in fifteen subjects at least 5 years of age whose assessments were not limited by contractures. Tables 8 and 9 present the 6 month and change from baseline data for upper and lower extremity strength assessments. There were no statistically significant differences between the two treatment groups (Table 10).

**Table 8:** Myometry of upper extremity by treatment arm – 6 month values and change from baseline.

	Placebo^1^	CARNIVAL^2^
Characteristic	N = 31	N = 30
Right Elbow 6 Month Value (kg)		
N	7	8
Mean	**2.17**	**1.55**
SD	1.24	0.56
Median	1.45	1.35
Range	1.20–4.17	0.98–2.72
Right Elbow Change from Baseline (kg)		
N	7	7
Mean	**−0.04**	**0.41**
SD	0.45	0.45
Median	−0.07	0.40
Range	−0.87–0.53	−0.25–0.97
Left Elbow 6 Month Value (kg)		
N	7	8
Mean	**2.21**	**1.47**
SD	1.31	0.42
Median	1.65	1.50
Range	0.95–4.30	0.83–2.23
Left Elbow Change from Baseline (kg)		
N	7	7
Mean	**0.11**	**0.22**
SD	0.65	0.33
Median	0.02	0.21
Range	−1.12–0.99	−0.17–0.83
Upper Extremity 6 Month Value (kg)		
N	7	8
Mean	**4.38**	**3.02**
SD	2.53	0.94
Median	3.10	2.88
Range	2.15–8.07	1.81–4.96
Upper Extremity Change from Baseline (kg)		
N	7	7
Mean*	**0.07**	**0.64**
SD	1.04	0.60
Median	−0.04	0.61
Range	−1.99–1.03	0.01–1.81

1 =  placebo group received matched placebo for both medications, L-carnitine and VPA.

2 = active treatment group received both L-carnitine and VPA.

*Difference  = 0.566. Upper confidence level difference 1.581. Lower confidence level difference −0.450.

**Table 9:** Myometry of lower extremity by treatment arm - 6 month values and change from baseline.

	Placebo^1^	CARNIVAL^2^
Characteristic	N = 31	N = 30
Right Knee 6 Month Value (kg)		
N	6	7
Mean	**0.87**	**1.00**
SD	0.42	0.77
Median	0.84	1.13
Range	0.40–1.47	0.00–2.0
Right Knee Change from Baseline (kg)		
N	4	6
Mean	**−0.39**	**0.24**
SD	1.13	0.40
Median	−0.05	0.18
Range	−2.0–0.54	−0.2–0.9
Left Knee 6 Month Value (kg)		
N	6	8
Mean	**0.78**	**1.02**
SD	0.41	0.60
Median	0.72	0.90
Range	0.40–1.50	0.20–1.77
Left Knee Change from Baseline (kg)		
N	4	6
Mean	**−0.46**	**0.31**
SD	1.11	0.45
Median	0.07	0.17
Range	−2.13–0.14	−0.20–1.03
Lower Extremity 6 Month Value (kg)		
N	6	8
Mean	**1.65**	**1.90**
SD	0.82	1.38
Median	1.57	1.67
Range	0.85–2.97	0.20–3.77
Lower Extremity Change from Baseline (kg)		
N	4	6
Mean*	**−0.85**	**0.55**
SD	2.22	0.83
Median	0.37	0.26
Range	−4.13–0.64	−0.27–1.93

1 =  placebo group received matched placebo for both medications, L-carnitine and VPA.

2 = active treatment group received both L-carnitine and VPA.

*Difference  = 1.40. Upper confidence level difference is 4.79, Lower confidence level difference is −1.98.

**Table 10:** Total myometry by treatment arm - 6 month values and change from baseline.

	Placebo	CARNIVAL
Characteristic	N = 31	N = 30
Total Myometry 6 Month Value (kilograms)		
N	8	8
Mean	**5.07**	**4.92**
SD	2.91	2.04
Median	4.52	4.60
Range	1.27–10.14	2.87–8.72
Total Myometry Change from Baseline (kilograms)		
N	8	7
Mean*	**−0.25**	**1.18**
SD	2.47	0.91
Median	0.65	0.70
Range	−6.12–1.41	0.52–2.91

1 =  placebo group received matched placebo for both medications, L-carnitine and VPA.

2 = active treatment group received both L-carnitine and VPA.

*Difference  = 1.431. Upper confidence level difference 3.550. Lower confidence level difference −0.689.

At 12 months, there were no statistically significant differences between groups initially assigned to placebo or to treatment. In the group receiving treatment for the full year, there was no continual improvement over the second six-month treatment period (data not shown). A generalized estimating equation model indicates that there is no period by arm effect (p = 0.73) and no BMI effect (p = 0.7983).

#### Impact of treatment on pulmonary function

PFTs were performed only in subjects five years of age and older (n = 24), thus limiting the power to observe any relationship with treatment. Treatment was not associated with changes in any of the PFT outcomes over the treatment period (data not shown).

#### Impact of treatment on quantitative measures of SMN mRNA in whole blood

Results of mRNA analysis at 6 months are in Table 11. There was no difference in actual baseline values or change from baseline values between the 2 treatment groups at 6 months. In addition, no difference was apparent between treatment groups through 12 months for either flSMN or Δ7 SMN. A mixed effect model analysis over the entire year indicated that baseline flSMN was highly predictive of flSMN at one year (p<0.0001), indicating very little change in flSMN. The correlation between Δ7 SMN baseline and Δ7 SMN at one year was less than flSMN, but still highly significant (p<0.0001). The minimal change over time and lack of correlation with baseline function provides little indication of a relationship between these mRNA parameters and outcome measures.

**Table 11:** SMN mRNA by treatment arm - 6 month values and change from baseline.

		Placebo^1^	CARNI-VAL^2^	p-value
flSMN	N	22	18	
	Mean	**2.01**	**2.47**	0.31
	SD	1.13	1.67	
	Min	0.58	0.53	
	Max	5.87	6.04	
Δ7 SMN	N	22	18	
	Mean	**6.76**	**7.02**	0.43
	SD	1.02	1.04	
	Min	4.49	4.92	
	Max	8.42	8.34	
Change from baseline flSMN	N	22	18	
	Mean	**0.00**	**0.03**	0.62
	SD	0.11	0.22	
	Min	−0.20	−0.37	
	Max	0.21	0.62	
Change from baseline Δ7 SMN	N	22	18	
	Mean	**−0.09**	**0.03**	0.62
	SD	0.82	0.81	
	Min	−2.19	−1.22	
	Max	1.78	2.56	

1 =  placebo group received matched placebo for both medications, L-carnitine and VPA.

2 = active treatment group received both L-carnitine and VPA.

SMN = Survival Motor Neuron, flSMN =  Full-length survival motor neuron mRNA levels, Δ7SMN = delta 7 SMN (missing exon 7) survival motor neuron mRNA levels.

### VPA trough levels

Eight participants (13%) were less than fully compliant with recommended VPA dosing, and eight participants (13%) were less than fully compliant with carnitine during one or more study visits during phase 1. Mean overnight VPA trough level in Group 2 (subjects receiving treatment) during phase 1 was 58.5 +/− 63.3 SD (16.8 to 101.0, range), which falls within the targeted trough level range of 50–100 mg/dL. VPA levels at 6 months were not associated with change in MHFMS.

### Adverse Events

Adverse events occurred in 58% of those receiving placebo and in 77% of treated subjects during the randomized treatment phase. Although there were no statistically significant differences between the treatment groups for adverse events, numbers of subjects reporting adverse events, serious adverse events, treatment-related adverse events, and severe adverse events were all greater in the treatment group (Table 12). A detailed listing of all adverse events during the treatment phase is presented in Supplemental Table S8. Gastrointestinal symptoms and respiratory symptoms were more frequent in the treatment group. Four subjects (13%) on active treatment had a severe adverse event, most commonly pneumonia (Table 13). Treatment-related adverse events were nearly equal between groups. There was no pattern in the placebo group, while gastrointestinal AE were the most common in the treatment group. Overall adverse events for the entire 12- month period in each treatment group are presented in Supplemental Table S9.

**Table 12:** Overall crude incidence of adverse events by treatment arm - baseline to 6 months (Phase I).*

	Group 1 Placebo^1^	Group 2 CARNI-VAL^2^	
Endpoint	N = 31 (%)	N = 30 (%)	p-value
Subjects Reporting ≥ One Adverse Event	18 (58)	23 (77)	0.1737
Subjects Reporting ≥ One SAE	1 (3)	4 (13)	0.1953
Subjects Reporting ≥ One Treatment-Related Adverse Event	9 (29)	11 (36)	0.5921
Subjects Reporting ≥ One Severe AE	2 (6)	6 (20)	0.1466
Subjects Reporting ≥ One Treatment-Related SAE	0 (0)	0 (0)	

1 =  placebo group received matched placebo for both medications, L-carnitine and VPA.

2 = active treatment group received both L-carnitine and VPA.

SAE = Serious Adverse Event.

*Relative risks (RR) are as follows for the categories included in this table: RR for 1 AE  = 1.32 (0.92, 1.89); RR for 1SAE  = 4.13 (0.49,34.89); RR for 1 treatment-related AE  = 1.26 (0.61,2.60); RR for 1 severe AE  = 3.10 (0.68, 14.17).

**Table 13:** Severe adverse events during Phase 1 by treatment group.

System Organ Class/Preferred Term (MedDRA)	Placebo^1^ N = 31 n (%)	CARNI-VAL^2^ N = 30 n (%)
**Gastrointestinal Disorders**	0 (0)	2 (7)
Vomiting	0 (0)	2 (7)
**General Disorders and Administration Site Conditions**	0 (0)	2 (7)
Pyrexia	0 (0)	2 (7)
**Infections and Infestations**	1 (3)	1 (3)
Upper Respiratory Infection	1 (3)	0 (0)
Pneumonitis	0 (0)	1 (3)
**Respiratory, Thoracic and Mediastinal Disorders**	0 (0)	5 (17)
Cough	0 (0)	1 (3)
Pneumonia	0 (0)	3 (10)
Tachypnoea	0 (0)	1 (3)
Nasal Congestion	0 (0)	1 (3)
**Metabolism and Nutrition Disorders**	0 (0)	1 (3)
Dehydration	0 (0)	1 (3)

1 =  placebo group received matched placebo for both medications, L-carnitine and VPA.

2 = active treatment group received both L-carnitine and VPA.

medDRA = Medical Dictionary for Regulatory Activities.

## Discussion

We chose to perform a placebo-controlled study of VPA in non-ambulatory children with SMA based on encouraging preliminary observations of improved motor function during an open label study [27]. Our choice to target children ages 2–8 years reflected a compromise between concerns regarding the lower limit of age where reliable assessment of motor function was possible versus concerns that improvement in older children may be constrained by other factors that would confound assessment of an SMN-enhanced treatment effect. A combined treatment regimen of VPA and carnitine was selected to avoid a potential confounding effect of carnitine depletion, because we had previously demonstrated an apparent susceptibility for more rapid depletion of carnitine with VPA treatment in this population. [27] Overall, enrollment was targeted towards a more homogeneous younger cohort of subjects who theoretically might be more likely to respond to therapy.

We did not detect a statistically significant improvement after six months of treatment in our primary outcome measure - the change in overall gross motor function assessed with the MHFMS. In addition, we did not detect a significant change in secondary endpoints of strength and function, which included assessment of maximum upper and lower extremity strength via hand-held myometry and PFTs. Adverse event frequency, although not statistically different between groups, was somewhat higher in the treatment group, particularly with regard to gastrointestinal and respiratory symptoms. Although we did not observe any clinical or laboratory evidence of serious hematologic or hepatic toxicity in this study, excessive weight gain was clearly more prevalent in the active treatment group. DEXA measurements confirmed that the associated weight gain was due largely to an increase in total body fat mass in the absence of an increase in lean mass.

Given the increased fat mass in the treatment group compared to the placebo group during the phase 1 period, it is notable that we did not observe a decline in gross motor function in the treatment group as a whole. As in the open label study [27], weight gain was not uniform across the population. Non-ambulatory subjects greater than five years of age, and those having a higher BMI at baseline, were at greatest risk. Children with the greatest improvement in gross motor function as assessed by the MHFMS gained less fat mass than did those with stable or worsening MHFMS scores. The fact that the MHFMS did not decline in light of substantial weight gain in the active treatment group suggests that VPA may indeed have a modest yet measurable biologic effect that is outweighed by its confounding effect of weight gain. Increases in fat mass may not always be deleterious in this population, where both over- and under-nourished states are common. Some children may benefit from the improved nutritional intake stimulated by an effect of VPA on appetite, perhaps putting them into a more favorable anabolic state. However, in other cases, due to increased gastrointestinal symptoms related to VPA and/or L-carnitine, weight loss could be a negative confounding factor.

Although no treatment effect was detected in the primary outcome measure as assessed by the MHFMS, a post hoc analysis demonstrated a statistically significant benefit in the youngest cohort of children, those two to three years of age. In addition, individuals who received the active treatment over a full year showed modestly improved function, gaining just over 2 points on the MHFMS, although this did not reach statistical or obvious functional significance. Age proved to be a significant factor as to whether or not subjects demonstrated an increase in MHFMS scores in association with treatment. We have previously demonstrated that increased age is associated with increased severity of denervation, so it is not surprising that the youngest non-ambulatory SMA children appear more likely to demonstrate a benefit with intervention [12]. Duration of treatment also appeared to be a factor and will need to be carefully considered in the design of future clinical trials in non-ambulatory SMA subjects. The ideal trial duration clearly depends on the expected mechanism of the treatment to be studied and relies heavily on the primary outcome measure chosen. This study affirms the value of the MHFMS in the multicenter pediatric clinical trial setting as a simple, reliable and easy to use outcome measure for documenting gross motor function in children as young as two years of age. However, if reinnervation is considered the most likely mechanism for a given therapeutic intervention, a trial duration of at least one year or longer may be more ideal to prove a treatment benefit using the MHFMS.

Possible disease-related biomarkers assessed in this study included quantitative SMN mRNA and maximum ulnar CMAP. Levels of flSMN and Δ7 SMN mRNA did not change with VPA. In contrast, VPA-response has been observed in SMA patient cell lines (16,17), one-third of SMA subjects (18) and in spinal cord of type III-like SMA mice (24). This result suggests that SMN expression in blood may not be a good surrogate biomarker to track VPA response. Because VPA targets are not specific, VPA response in SMA subjects may be much more complex than simply altering SMN expression. Indeed, recent data in a mild SMA model suggests that VPA may function by increasing SMN mRNA and protein, decreasing apoptosis and enhancing neuroprotection (24). Alternatively, whole blood may not be the appropriate cell type to detect changes in SMN expression, or such changes may be too small to be detected by relative quantification that relies on endogenous controls to normalize data. Finally, the assay we used has limitations, because it is not based on absolute quantification of SMN mRNA.

The strength of correlation between maximum CMAP amplitude and our primary outcome measure, the MHFMS, suggests that in non-ambulatory patients with SMA the CMAP may be a robust surrogate outcome measure for assessing treatment interventions if the putative mechanism of effect is to enhance innervated muscle mass by reinnervation or other trophic effects on nerve or muscle. However, baseline severity of denervation overall in a given cohort will profoundly impact whether or not improvement is anticipated and should be a key consideration in any trial design incorporating this as an outcome measure, as ceiling and floor effects may limit the usefulness of this assay in those of greater or lesser strength or age.

In summary, this study demonstrated no benefit from six months of treatment with VPA and L-carnitine in a young non-ambulatory cohort of subjects with SMA. Any modest biologic impact of VPA on individual outcome measures over the duration of treatment in this study was clearly outweighed by weight gain. This trial confirmed observations made in the open label study that the effects of age and weight gain with VPA are critical confounding factors that will have to be considered in the design of future clinical trials. Whether or not a subset of younger non-ambulatory SMA subjects might benefit from VPA treatment early in the course of their disease over a longer treatment period remains an outstanding question. Treatment intervention with VPA in non-ambulatory SMA children outside of a clinical study should be discouraged given the lack of clear benefit from this trial, and the potentially serious adverse events associated with VPA treatment. However, these results should be interpreted in the context of the limited treatment duration examined, an issue which should be carefully considered in the design of future clinical trials based on proposed mechanism of therapeutic action. We anxiously await the completion of ongoing studies of VPA in an ambulatory adult population (the VALIANT trial, clinicaltrials.gov ID NCT00481013) to help us further expand our knowledge about possible biologic impact of VPA in subjects with SMA. The ability to obtain strength data from the entire cohort in the VALIANT trial should help to answer outstanding questions about potential biologic effects of VPA in subjects with SMA, and whether this agent merits further study as a therapeutic agent in this disorder.

## Supporting Information

Table S1 Baseline Body Mass by Treatment Arm.
(0.05 MB DOC)

Table S2 Body Composition at Baseline by Treatment Arm.
(0.04 MB DOC)

Table S3 Pulmonary Function Testing at Baseline by Treatment Arm.
(0.06 MB DOC)

Table S4 CMAP Values at Baseline by Treatment Arm.
(0.04 MB DOC)

Table S5 Myometry Scores at Baseline by Treatment Arm.
(0.06 MB DOC)

Table S6 PedsQL Parent Proxy Sub scores at Baseline by Treatment Group.
(0.05 MB DOC)

Table S7 PedsQL Child Assessment at Baseline by Treatment Arm.
(0.05 MB DOC)

Table S8 Adverse events occuring during Phase I arm.
(0.10 MB DOC)

Table S9 Overall adverse events during all 12 months.
(0.08 MB DOC)

Protocol S1 Protocol Summary.
(0.20 MB DOC)

Checklist S1 CONSORT Statement 2001 Checklist.
(0.19 MB DOC)
