# Supplementary material for: SMA CARNI-VAL Trial Part I: Double-Blind, Randomized, Placebo-Controlled Trial of L-Carnitine and Valproic Acid in Spinal Muscular Atrophy
Source: PLoS One. 2010 Aug 19;5(8):e12140. doi: 10.1371/journal.pone.0012140 (PMC2924376; doi:10.1371/journal.pone.0012140)
Supplement: Table S1 — Baseline Body Mass by Treatment Arm. (0.05 MB DOC) [file pone.0012140.s001.doc]

| **Supplemental Table S1. Baseline Body Mass by Treatment Arm** | | | |
| --- | --- | --- | --- |
|  | Placebo^1^ | CARNIVAL^2^ | Total |
| Characteristic | N=31 | N=30 | N=61 |
| Weight (kg) |  | | |
| Mean | 16.5 | 14.7 | 15.6 |
| SD | 4.8 | 4.9 | 4.9 |
| Median | 15.2 | 13.4 | 14.0 |
| Range | 11.4-30.5 | 9.1-32.4 | 9.1-32.4 |
| Height (cm) |  | | |
| Mean | 103.5 | 100.0 | 101.8 |
| SD | 11.6 | 13.7 | 12.7 |
| Median | 102.0 | 96.7 | 100.5 |
| Range | 88.5-127.0 | 82.7-138.0 | 82.7-138.0 |
| BMI |  | | |
| Mean | 15.1 | 14.4 | 14.7 |
| SD | 2.0 | 1.7 | 1.8 |
| Median | 14.9 | 14.1 | 14.5 |
| Range | 10.2-18.9 | 11.5-18.2 | 10.2-18.9 |
| BMI Z-score |  | | |
| Mean | -1.17 | -1.76 | -1.46 |
| SD | 2.32 | 2.03 | 2.18 |
| Median | -0.89 | -1.69 | -1.34 |
| Range | -10.4-1.82 | -8.06-1.77 | -10.4-1.82 |
| BMI Percent |  | | |
| Mean | 33.9 | 21.5 | 27.3 |
| SD | 35.0 | 28.9 | 32.4 |
| Median | 18.7 | 4.6 | 9.0 |
| Range | <0.001-96.6 | <0.001-96.2 | <0.001-96.6 |

1= placebo group received matched placebo for both medications, L-carnitine and VPA

2=active treatment group received both L-carnitine and VPA

BMI= Body Mass Index
