# Supplementary material for: SMA CARNI-VAL Trial Part I: Double-Blind, Randomized, Placebo-Controlled Trial of L-Carnitine and Valproic Acid in Spinal Muscular Atrophy
Source: PLoS One. 2010 Aug 19;5(8):e12140. doi: 10.1371/journal.pone.0012140 (PMC2924376; doi:10.1371/journal.pone.0012140)
Supplement: Table S2 — Body Composition at Baseline by Treatment Arm. (0.04 MB DOC) [file pone.0012140.s002.doc]

| **Supplemental Table S2. Body Composition at Baseline by Treatment Arm** | | | |
| --- | --- | --- | --- |
|  | Placebo^1^ | CARNI-VAL^2^ | Total |
| Characteristic | N=16 | N=13 | N=29 |
| Total body bone mineral density (BMD), grams/cm^2^ | | | |
| Mean | 0.81 | 0.78 | 0.80 |
| SD | 0.12 | 0.16 | 0.14 |
| Median | 0.83 | 0.85 | 0.83 |
| Range | 0.49-0.94 | 0.42-0.93 | 0.42-0.94 |
| Total body bone mineral content (BMC), grams | | | |
| Mean | 519.9 | 474.5 | 499.5 |
| SD | 274.0 | 210.5 | 244.4 |
| Median | 413.8 | 401.5 | 401.5 |
| Range | 224.0-1109 | 223-895 | 223-1109 |
| Lean Mass (grams) | | | |
| Mean | 7720.4 | 6215.1 | 7045.4 |
| SD | 2698.5 | 2712.5 | 2763.1 |
| Median | 7784.0 | 4896.0 | 6299 |
| Range | 4943.0-15300.0 | 3710-13128 | 3710-15300 |
| Fat Mass (grams) | | | |
| Mean | 7658.2 | 6735 | 7244.4 |
| SD | 5707.9 | 3803.9 | 4886.0 |
| Median | 4844 | 6964 | 5546 |
| Range | 1700-19716 | 1400-13790 | 1400-19716 |

1= placebo group received matched placebo for both medications, L-carnitine and VPA

2=active treatment group received both L-carnitine and VPA
