# Supplementary material for: SMA CARNI-VAL Trial Part I: Double-Blind, Randomized, Placebo-Controlled Trial of L-Carnitine and Valproic Acid in Spinal Muscular Atrophy
Source: PLoS One. 2010 Aug 19;5(8):e12140. doi: 10.1371/journal.pone.0012140 (PMC2924376; doi:10.1371/journal.pone.0012140)
Supplement: Table S3 — Pulmonary Function Testing at Baseline by Treatment Arm. (0.06 MB DOC) [file pone.0012140.s003.doc]

| **Supplemental Table S3. Pulmonary Function Testing at Baseline by Treatment Arm** | | | |
| --- | --- | --- | --- |
|  | Placebo^1^ | CARNIVAL^2^ | Total |
| Characteristic | N=31 | N=30 | N=61 |
| Forced Vital Capacity (FVC, Liters) | | | |
| N | 10 | 14 | 24 |
| Mean | 1.17 | 1.25 | 1.22 |
| SD | 0.37 | 0.99 | 0.78 |
| Median | 1.09 | 1.07 | 1.09 |
| Range | 0.64-2.0 | 0.55-4.5 | 0.55-4.5 |
| FVC % Predicted | | | |
| N | 11 | 13 | 24 |
| Mean | 79.3 | 75.0 | 76.9 |
| SD | 15.8 | 15.5 | 15.4 |
| Median | 80.0 | 78.0 | 79.0 |
| Range | 56.0-105.0 | 52.0-107.0 | 52.0-107.0 |
| Forced Expiratory Volume in One Second (FEV1, Liters) | | | |
| N | 9 | 13 | 22 |
| Mean | 0.99 | 0.85 | 0.91 |
| SD | 0.23 | 0.32 | 0.29 |
| Median | 0.94 | 0.86 | 0.91 |
| Range | 0.64-1.32 | 0.43-1.31 | 0.43-1.32 |
| FEV1 % Predicted | | | |
| N | 9 | 13 | 22 |
| Mean | 83.6 | 73.4 | 77.6 |
| SD | 16.1 | 19.9 | 18.7 |
| Median | 85 | 68 | 76 |
| Range | 60-112 | 39-108 | 39-112 |
| Maximum Inspiratory Pressure (MIP, cm H_2_O) | | | |
| N | 10 | 14 | 24 |
| Mean | 51.3 | 45.6 | 48.0 |
| SD | 10.9 | 16.7 | 14.5 |
| Median | 51.0 | 42.4 | 50.0 |
| Range | 32-71 | 19-81 | 19-81 |
| MIP % Predicted | | | |
| N | 7 | 8 | 15 |
| Mean | 73.6 | 57.5 | 65 |
| SD | 22.8 | 25.9 | 25.1 |
| Median | 75.0 | 52.0 | 65 |
| Range | 41-100 | 19-100 | 19-100 |
| Maximum Expiratory Pressure (MEP, cm H_2_0) | | | |
| N | 10 | 14 | 24 |
| Mean | 48.3 | 36.5 | 41.4 |
| SD | 19.9 | 14.9 | 17.7 |
| Median | 46.0 | 30.8 | 38.5 |
| Range | 24.5-93.0 | 21.0-71.0 | 21.0-93.0 |
| MEP %Predicted | | | |
| N | 7 | 8 | 15 |
| Mean | 61.9 | 38.6 | 49.5 |
| SD | 35.2 | 33.1 | 35.0 |
| Median | 67.0 | 67.0 | 27 |
| Range | 17-100 | 17-100 | 17-100 |

1= placebo group received matched placebo for both medications, L-carnitine and VPA

2=active treatment group received both L-carnitine and VPA
