# Supplementary material for: SMA CARNI-VAL Trial Part I: Double-Blind, Randomized, Placebo-Controlled Trial of L-Carnitine and Valproic Acid in Spinal Muscular Atrophy
Source: PLoS One. 2010 Aug 19;5(8):e12140. doi: 10.1371/journal.pone.0012140 (PMC2924376; doi:10.1371/journal.pone.0012140)
Supplement: Table S4 — CMAP Values at Baseline by Treatment Arm. (0.04 MB DOC) [file pone.0012140.s004.doc]

| **Supplemental table S4. CMAP Values at Baseline by Treatment Arm** | | | |
| --- | --- | --- | --- |
|  | Placebo^1^ | CARNIVAL^2^ | Total |
| Characteristic | N=31 | N=30 | N=61 |
| Maximum ulnar Compound Muscle Action Potential Amplitude (CMAP, mV) | | | |
| N | 23 | 21 | 44 |
| Mean | 2.28 | 2.93 | 2.33 |
| SD | 1.88 | 1.56 | 1.71 |
| Median | 1.91 | 2.20 | 2.06 |
| Range | 0.50-7.66 | 0.50-6.66 | 0.50-7.66 |
| Maximum ulnar CMAP area (mVus) | | | |
| N | 23 | 21 | 44 |
| Mean | 5.46 | 5.45 | 5.45 |
| SD | 5.03 | 4.23 | 4.61 |
| Median | 3.60 | 4.60 | 4.24 |
| Range | 0.70-19.71 | 1.30-16.78 | 0.70-19.71 |

1= placebo group received matched placebo for both medications, L-carnitine and VPA

2=active treatment group received both L-carnitine and VPA
