# Supplementary material for: SMA CARNI-VAL Trial Part I: Double-Blind, Randomized, Placebo-Controlled Trial of L-Carnitine and Valproic Acid in Spinal Muscular Atrophy
Source: PLoS One. 2010 Aug 19;5(8):e12140. doi: 10.1371/journal.pone.0012140 (PMC2924376; doi:10.1371/journal.pone.0012140)
Supplement: Table S5 — Myometry Scores at Baseline by Treatment Arm. (0.06 MB DOC) [file pone.0012140.s005.doc]

| **Supplemental Table S5. Myometry Scores at Baseline by Treatment Arm** | | | |
| --- | --- | --- | --- |
|  | Placebo^1^ | CARNI-VAL^2^ | Total |
| Characteristic | N=31 | N=30 | N=61 |
| Right Elbow (kilograms) | | | |
| N | 9 | 9 | 18 |
| Mean | 2.35 | 1.15 | 1.75 |
| SD | 1.05 | 0.55 | 1.02 |
| Median | 2.32 | 1.20 | 1.55 |
| Range | 1.02-4.20 | 0.00-1.77 | 0.00-4.20 |
| Left Elbow (kilograms) | | | |
| N | 8 | 9 | 17 |
| Mean | 2.25 | 1.21 | 1.70 |
| SD | 1.05 | 0.65 | 0.99 |
| Median | 2.17 | 1.17 | 1.45 |
| Range | 0.97-3.80 | 0.00-2.40 | 0.00-3.80 |
| Upper Extremity TOTAL (kilograms) | | | |
| N | 9 | 9 | 18 |
| Mean | 4.35 | 2.36 | 3.36 |
| SD | 2.07 | 1.11 | 1.91 |
| Median | 3.25 | 2.37 | 2.71 |
| Range | 2.27-7.07 | 0.00-4.17 | 0.00-7.07 |
| Right Knee (kilograms) | | | |
| N | 7 | 8 | 15 |
| Mean | 0.96 | 0.91 | 0.93 |
| SD | 0.97 | 1.11 | 1.01 |
| Median | 0.92 | 0.45 | 0.50 |
| Range | 0.00-2.97 | 0.00-3.07 | 0.00-3.07 |
| Left Knee (kilograms) | | | |
| N | 5 | 8 | 13 |
| Mean | 1.14 | 0.77 | 0.91 |
| SD | 1.17 | 0.79 | 0.92 |
| Median | 0.73 | 0.62 | 0.67 |
| Range | 0.00-3.03 | 0.00-1.97 | 0.00-3.03 |
| Lower Extremity TOTAL (kilograms) | | | |
| N | 7 | 8 | 15 |
| Mean | 1.77 | 1.69 | 1.73 |
| SD | 2.05 | 1.85 | 1.87 |
| Median | 1.57 | 0.83 | 1.07 |
| Range | 0.00-6.01 | 0.00-4.89 | 0.00-6.01 |
| Myometry TOTAL (kilograms) | | | |
| N | 10 | 9 | 19 |
| Mean | 5.2 | 3.9 | 4.5 |
| SD | 3.2 | 2.6 | 2.9 |
| Median | 4.1 | 2.9 | 3.4 |
| Range | 1.6-11.1 | 0.0-8.2 | 0.0-11.1 |

1= placebo group received matched placebo for both medications, L-carnitine and VPA

2=active treatment group received both L-carnitine and VPA
