# Supplementary material for: SMA CARNI-VAL Trial Part I: Double-Blind, Randomized, Placebo-Controlled Trial of L-Carnitine and Valproic Acid in Spinal Muscular Atrophy
Source: PLoS One. 2010 Aug 19;5(8):e12140. doi: 10.1371/journal.pone.0012140 (PMC2924376; doi:10.1371/journal.pone.0012140)
Supplement: Table S6 — PedsQL Parent Proxy Sub scores at Baseline by Treatment Group. (0.05 MB DOC) [file pone.0012140.s006.doc]

| **Supplemental Table S6. PedsQL Parent Proxy Sub scores at Baseline by Treatment Group** | | | |
| --- | --- | --- | --- |
|  | Placebo^1^ | CARNI-VAL^2^ | Total |
| Characteristic | N=31 | N=30 | N=61 |
| Physical Function | | | |
| N | 30 | 30 | 60 |
| Mean | 36.9 | 30.8 | 33.8 |
| SD | 22.0 | 18.9 | 20.6 |
| Median | 29.7 | 26.6 | 28.1 |
| Range | 9.4-81.2 | 12.5-87.5 | 9.4-87.5 |
| Emotional Function | | | |
| N | 30 | 30 | 60 |
| Mean | 72.5 | 70.8 | 71.7 |
| SD | 14.1 | 14.9 | 14.4 |
| Median | 72.5 | 70 | 70 |
| Range | 40-100 | 45-100 | 40-100 |
| Social Function | | | |
| N | 30 | 30 | 60 |
| Mean | 60.3 | 61.3 | 60.8 |
| SD | 16.7 | 16.6 | 16.5 |
| Median | 57.5 | 65 | 60 |
| Range | 25-90 | 30-90 | 25-90 |
| School Function | | | |
| N | 24 | 25 | 49 |
| Mean | 65.4 | 67.5 | 66.5 |
| SD | 18.5 | 13.7 | 16.1 |
| Median | 66.7 | 66.7 | 66.7 |
| Range | 25-100 | 40-100 | 25-100 |
| Psychosocial | | | |
| N | 30 | 30 | 60 |
| Mean | 68.3 | 68.6 | 68.4 |
| SD | 14.4 | 12.2 | 13.2 |
| Median | 69.0 | 67 | 68.5 |
| Range | 31.2-97.5 | 45.4-100 | 31.2-100 |
| Total QOL | | | |
| N | 30 | 30 | 60 |
| Mean | 54.9 | 52.6 | 53.8 |
| SD | 13.7 | 11.6 | 12.7 |
| Median | 53.7 | 52.6 | 52.6 |
| Range | 20-77.8 | 35-86.7 | 20-86.7 |

1= placebo group received matched placebo for both medications, L-carnitine and VPA

2=active treatment group received both L-carnitine and VPA

QOL=Quality of Life
