# Supplementary material for: SMA CARNI-VAL Trial Part I: Double-Blind, Randomized, Placebo-Controlled Trial of L-Carnitine and Valproic Acid in Spinal Muscular Atrophy
Source: PLoS One. 2010 Aug 19;5(8):e12140. doi: 10.1371/journal.pone.0012140 (PMC2924376; doi:10.1371/journal.pone.0012140)
Supplement: Table S7 — PedsQL Child Assessment at Baseline by Treatment Arm. (0.05 MB DOC) [file pone.0012140.s007.doc]

| **Supplemental Table S7. PedsQL Child Assessment at Baseline by Treatment Arm** | | | |
| --- | --- | --- | --- |
|  | Placebo^1^ | CARNI-VAL^2^ | Total |
| Characteristic | N=31 | N=30 | N=61 |
| Physical Function | | | |
| N | 12 | 10 | 22 |
| Mean | 46.7 | 49.1 | 47.8 |
| SD | 9.5 | 19.0 | 14.3 |
| Median | 50 | 46.9 | 50 |
| Range | 25-62.5 | 25-84.4 | 25-84.4 |
| Emotional Function | | | |
| N | 12 | 10 | 22 |
| Mean | 66.7 | 58.5 | 63.0 |
| SD | 16.1 | 11.6 | 14.5 |
| Median | 60 | 55 | 60 |
| Range | 50-100 | 45-80 | 45-100 |
| Social Function | | | |
| N | 11 | 10 | 21 |
| Mean | 60 | 66 | 62.9 |
| SD | 17.9 | 16.5 | 17.1 |
| Median | 60 | 75 | 70 |
| Range | 20-80 | 40-80 | 20-80 |
| School Function | | | |
| N | 11 | 10 | 21 |
| Mean | 62.0 | 67.5 | 64.6 |
| SD | 19.4 | 10.9 | 15.8 |
| Median | 70 | 65 | 70 |
| Range | 10-80 | 50-80 | 10-80 |
| Psychosocial | | | |
| N | 12 | 10 | 22 |
| Mean | 62.5 | 64 | 63.2 |
| SD | 12.3 | 9.4 | 10.9 |
| Median | 66.7 | 65 | 66.7 |
| Range | 33.3-76.7 | 50-75 | 33.3-76.7 |
| Total QOL | | | |
| N | 12 | 10 | 22 |
| Mean | 57.0 | 58.8 | 57.8 |
| SD | 7.5 | 11.4 | 9.3 |
| Median | 57.5 | 62 | 58.7 |
| Range | 43.5-67.4 | 42.4-77.2 | 42.4-77.2 |

1= placebo group received matched placebo for both medications, L-carnitine and VPA

2=active treatment group received both L-carnitine and VPA

QOL=Quality of Life
