# Supplementary material for: SMA CARNI-VAL Trial Part I: Double-Blind, Randomized, Placebo-Controlled Trial of L-Carnitine and Valproic Acid in Spinal Muscular Atrophy
Source: PLoS One. 2010 Aug 19;5(8):e12140. doi: 10.1371/journal.pone.0012140 (PMC2924376; doi:10.1371/journal.pone.0012140)
Supplement: Table S8 — Adverse events occuring during Phase I arm. (0.10 MB DOC) [file pone.0012140.s008.doc]

| **Supplemental Table S8. Adverse events occurring during Phase I arm.** | | | |
| --- | --- | --- | --- |
| **System Organ Class/ Preferred Term (MedDRA)** | **Placebo^1^ N=31 n (%)** | **CARNI-VAL^2^ N=30 n (%)** | **p-value** |
| **Gastrointestinal Disorders** | 6 (19) | 12 (40) | 0.0971 |
| Vomiting | 6 (19) | 6 (20) |  |
| Abdominal Pain Upper | 2 (6) | 3 (10) |  |
| Nausea | 2 (6) | 4 (13) |  |
| Constipation | 1 (3) | 0 (0) |  |
| Dry Mouth | 1 (3) | 0 (0) |  |
| Diarrhea | 0 (0) | 2 (7) |  |
| Gastroesophageal Reflux Disease | 0 (0) | 1 (3) |  |
| **General Disorders and Administration Site Conditions** | 6 (19) | 5 (17) |  |
| Pyrexia | 4 (13) | 5 (17) |  |
| Fatigue | 2 (6) | 0 (0) |  |
| **Immune System Disorders** | 4(13) | 4 (13) |  |
| Dermatitis Allergic | 3 (13) | 2 (7) |  |
| Multiple Allergies | 0 (0) | 2 (7) |  |
| Hypersensitivity | 1 (3) | 0 (0) |  |
| **Infections and Infestations** | 7 (23) | 11 (37) |  |
| Nasopharyngitis | 3 (10) | 3 (10) |  |
| Ear Infections | 2 (6) | 3 (10) |  |
| Urinary Tract Infection | 0 (0) | 3 (10) |  |
| Upper Respiratory Infection | 2 (6) | 1 ( 3) |  |
| Bronchitis | 0 (0) | 1 (3) |  |
| Pneumonitis | 1 (3) | 1 (3) |  |
| Sinusitis | 1 (3) | 0 (0) |  |
| Croup | 0 (0) | 1 (3) |  |
| Tinea Infection | 0 (0) | 1 (3) |  |
| **Respiratory, Thoracic and Mediastinal Disorders** | 4 (13) | 9 (30) | 0.1271 |
| Cough | 2 (6) | 7 (23) |  |
| Pneumonia | 2 (6) | 6 (20) |  |
| Pharyngitis Streptococcal | 1 (3) | 1 (3) |  |
| Dyspnea | 2 (6) | 0 (0) |  |
| Nasal Congestion | 2 (6) | 1 (3) |  |
| Tachypnoea | 0 (0) | 1 (3) |  |
| Choking Sensation | 0 (0) | 1 (3) |  |
| Pharyngeal Pain | 1 (3) | 0 (0) |  |
| **Injury, Poisoning and Procedural Complications** | 1 (3) | 0 (0) |  |
| Joint Sprain | 1 (3) | 0 (0) |  |
| **Investigations** | 4 (13) | 3 (10) |  |
| Weight Increased | 4 (13) | 3 (10) |  |
| **Musculoskeletal and Connective Tissue Disorders** | 1 (3) | 2 (7) |  |
| Arthralgia | 1 (3) | 0 (0) |  |
| Femur Fracture | 0 (0) | 1 (3) |  |
| Pain in Extremity | 0 (0) | 1 (3) |  |
| **Nervous System Disorders** | 3 (10) | 2 (7) |  |
| Headache | 1 (3) | 2 (7) |  |
| Tremor | 2 (6) | 0 (0) |  |
| **Psychiatric Disorders** | 5 (16) | 1 (3) | 0.1953 |
| Emotional Disorder of Childhood | 2 (6) | 0 (0) |  |
| Lethargy | 1 (3) | 0 (0) |  |
| Impulsive Behaviour | 0 (0) | 1 (3) |  |
| Irritability | 2 (6) | 1 (3) |  |
| Agitation | 1 (3) | 0 (0) |  |
| **Renal and Urinary Disorders** | 1 (3) | 0 (0) |  |
| Incontinence | 1 (3) | 0 (0) |  |
| **Ear and Laryrinth Disorders** | 0 (0) | 1 (3) |  |
| Otorrhoea | 0 (0) | 1 (3) |  |
| **Metabolism and Nutrition Disorders** | 3 (10) | 1 (3) |  |
| Increased Appetite | 2 (6) | 0 (0) |  |
| Decreased Appetite | 1 (3) | 0 (0) |  |
| Dehydration | 0 (0) | 1 (3) |  |
| **Skin and Subcutaneous Tissue Disorders** | 1 (3) | 1 (3) |  |
| Rash Papular | 1 (3) | 1 (3) |  |

1= placebo group received matched placebo for both medications, L-carnitine and VPA

2=active treatment group received both L-carnitine and VPA

medDRA=Medical Dictionary for Regulatory Activities
