# Supplementary material for: SMA CARNI-VAL Trial Part I: Double-Blind, Randomized, Placebo-Controlled Trial of L-Carnitine and Valproic Acid in Spinal Muscular Atrophy
Source: PLoS One. 2010 Aug 19;5(8):e12140. doi: 10.1371/journal.pone.0012140 (PMC2924376; doi:10.1371/journal.pone.0012140)
Supplement: Table S9 — Overall adverse events during all 12 months. (0.08 MB DOC) [file pone.0012140.s009.doc]

| **Supplemental Table S9. Overall adverse events during all 12 months.** | | |
| --- | --- | --- |
| **System Organ Class/  Preferred Term (MedDRA)** | **Group 1**  Placebo Phase I*  CARNI-VAL Phase II**  N=31  **n (%)** | **Group 2**  CARNI-VAL  Phases I* and II**  N=30  **n (%)** |
| **Gastrointestinal Disorders** | 12 (39) | 13 (43) |
| Vomiting | 11 (35) | 9 (30) |
| Abdominal Pain Upper | 4 (13) | 4 (13) |
| Nausea | 2 (6) | 4 (13) |
| Constipation | 1 (3) | 0 (0) |
| Dry Mouth | 1 (3) | 0 (0) |
| Diarrhea | 0 (0) | 2 (7) |
| Gastroesophageal Reflux Disease | 0 (0) | 1 (3) |
| **General Disorders and Administration Site Conditions** | 7 (23) | 8 (27) |
| Pyrexia | 6 (19) | 7 (23) |
| Chest Pain | 1 (3) | 0 (0) |
| Febrile Disorders | 0 (0) | 1 (3) |
| **Immune System Disorders** | 5 (16) | 4 (13) |
| Dermatitis Allergic | 4 (13) | 2 (7) |
| Multiple Allergies | 1 (3) | 2 (7) |
| **Infections and Infestations** | 12 (39) | 15 (50) |
| Ear Infections | 5 (16) | 4 (13) |
| Urinary Tract Infection | 1 (3) | 3 (10) |
| Upper Respiratory Infection | 2 (6) | 1( 3) |
| Bronchitis | 1 (3) | 2 (7) |
| Respiratory Syncytial Virus Infection | 1 (3) | 1 (3) |
| Pneumonitis | 1 (3) | 1 (3) |
| Sinus Infection | 1 (3) | 1 (3) |
| Croup | 0 (0) | 1 (3) |
| Gastroenteritis | 0 (0) | 1 (3) |
| **Respiratory, Thoracic and Mediastinal Disorders** | 17 (55) | 11 (37) |
| Nasopharyngitis | 8 (26) | 4 (13) |
| Cough | 8 (26) | 9 (30) |
| Pneumonia | 5 (16) | 9 (30) |
| Pharyngitis Streptococcal | 3 (10) | 2 (7) |
| Dyspnea | 1 (3) | 1 (3) |
| Nasal Congestion | 3 (10) | 2 (7) |
| Atelectasis | 1 (3) | 0 (0) |
| Rhinorrhoea | 1 (3) | 0 (0) |
| Pharyngeal Pain | 1 (3) | 0 (0) |
| **Injury, Poisoning and Procedural Complications** | 1 (3) | 1 (3) |
| Joint Sprain | 1 (3) | 1 (3) |
| **Investigations** | 6 (19) | 6 (20) |
| Weight Increased | 5 (16) | 5 (17) |
| Oxygen Saturation Decreased | 1 (3) | 0 (0) |
| Weight Loss | 0 (0) | 1 (3) |
| **Musculoskeletal and Connective Tissue Disorders** | 1 (3) | 4 (13) |
| Arthralgia | 1 (3) | 0 (0) |
| Femur Fracture | 0 (0) | 2 (7) |
| Patella Fracture | 0 (0) | 2 (7) |
| Foot Fracture | 0 (0) | 0 (0) |
| Forearm Fracture | 0 (0) | 0 (0) |
| Hand Fracture | 0 (0) | 0 (0) |
| Muscle Cramp | 0 (0) | 0 (0) |
| **Nervous System Disorders** | 3 (10) | 4 (13) |
| Headache | 1 (3) | 4 (13) |
| Migraine | 0 (0) | 0 (0) |
| Tremor | 1 (3) | 0 (0) |
| Lethargy | 1 (3) | 0 (0) |
| **Psychiatric Disorders** | 2 (6) | 0 (0) |
| Emotional Disorder of Childhood | 2 (6) | 0 (0) |
| **Renal and Urinary Disorders** | 2 (6) | 0 (0) |
| Incontinence | 1 (3) | 0 (0) |
| Increased Urination Frequency | 1 (3) | 0 (0) |

*Phase I = first six months treatment period in which subjects were randomized to receive either placebo for both VPA and L-carnitine or active treatment

**Phase II = intention to treat period in which all subjects receive active treatment

medDRA=Medical Dictionary for Regulatory Activities
