# Supplementary material for: SMA CARNI-VAL Trial Part I: Double-Blind, Randomized, Placebo-Controlled Trial of L-Carnitine and Valproic Acid in Spinal Muscular Atrophy
Source: PLoS One. 2010 Aug 19;5(8):e12140. doi: 10.1371/journal.pone.0012140 (PMC2924376; doi:10.1371/journal.pone.0012140)
Supplement: Protocol S1 — Protocol Summary. (0.20 MB DOC) [file pone.0012140.s010.doc]

# PROTOCOL SUMMARY

# *Version 3: 21 February 2007*

# **Multi-center Phase II Trial of Valproic Acid and Carnitine**

# **in Patients with Spinal Muscular Atrophy**

# **(SMA CARNI-VAL Trial)**

# Principal Investigator

# Kathryn J. Swoboda, M.D.

# University of Utah

# Department of Neurology

# 30 North 1900 East #3R210

# Salt Lake City, Utah 84132

# (801) 585-1676

# Co-Investigators

**Gyula Acsadi M.D., Ph.D.
Children's Hospital of Michigan**

**Department of Pediatrics and Neurology
Wayne State University, School of Medicine
3901 Beaubien Blvd.**

**Detroit, MI 48201**

**Thomas Crawford, M.D.**

**Johns Hopkins University**

**Department of Neurology**

**Jefferson Building, Room 123**

**600 North Wolfe Street**

**Baltimore, MD 21287**

**Guy D'Anjou, M.D., F.R.C.P.(c)**

**Neurologie Pediatrique, 5e bloc 4**

**Hopital Sainte-Justine**

**3175, Cote-Sainte-Catherine Road**

**Montreal, Quebec, Canada H3T-1C5**

**John T. Kissel, M.D.**

**Ohio State University**

**Department of Neurology**

**445 Means Hall**

**1654 Upham Drive**

**Columbus, Ohio 43210-1228**

**Mary K. Schroth, M.D.**

**Associate Professor of Pediatrics**

**University of Wisconsin Children’s Hospital**

**Pediatric Pulmonary & Cystic Fibrosis Center**

**600 Highland Avenue**

**Madison, WI 53792-9988**

# **Collaborators**

**Kristin Krosschell, P.T., M.A.**

**Department of Physical Therapy and Human Movement Sciences**

**Feinberg School of Medicine, Northwestern University**

**Suite 1100, 645 North Michigan Ave**

**Chicago, IL 60611**

**312-908-6790**

**Bernie LaSalle**

**General Clinical Research Center**

**University of Utah**

**30 North 1900 East #4R210**

**Salt Lake City, UT 84132-2411**

**Joanne Maczulski, O.T.R./L., M.A.**

**537 West Roscoe, #2N**

**Chicago, IL 60657**

**Thomas Prior, Ph.D.**

**Molecular Pathology**

**173 Hamilton Hall**

**1645 Neil Avenue**

**Columbus, OH 43210**

**Charles Scott, Ph.D.**

**Director, Children’s Hospital of Philadelphia**

**Biostatistics and Data Management**

**3535 Market Street 10^th^ Floor**

**Suite 1032 and 1035**

**Philadelphia, PA 19104**

**Louise R. Simard, Ph.D.**

**Associate Professor**

**Centre de Recherche de l'Hôpital Sainte-Justine**

**3175 Côte Sainte-Catherine Road**

**Montréal, Québec, Canada H3T 1C5**

**Background and Introduction**

Spinal muscular atrophy (SMA) is an autosomal recessive motor neuron disease characterized by diffuse weakness and muscular atrophy. It is one of the most common neuromuscular conditions, and a leading cause of hereditary infant mortality ^1-6^. Patients most frequently present in infancy and early childhood, but the clinical spectrum of onset and severity is broad, ranging from the infant with congenital joint contractures and ventilator dependence to adult onset of proximal muscle weakness^7^. This clinical heterogeneity increases the inherent challenges in clinical trial design for the evaluation of promising new therapies. Our work and that of others have demonstrated that SMA is a progressive motor neuron disease, as reflected by loss of function as well as maximum compound motor action potential amplitudes and motor unit number estimates in a distally innervated hand muscle over time ^8^. Disease progression can occur rapidly in SMA type 1 infants prior to reaching a stable albeit significantly weak endpoint, while patients with SMA types 2 and 3 often demonstrate a more slowly progressive course, with stable function preserved over many years. As an important prelude to this current proposal, pilot studies at the University of Utah have established stability of functional outcome measures over at least a six month time period in a cohort of non-ambulatory sitters and ambulatory SMA type 2 and 3 children.^9^

Homozygous deletion of the survival motor neuron 1 (SMN1) gene is found in more than 94% of SMA cases regardless of subtype, and plays a primary role in disease pathogenesis ^10-12^. The SMN1 gene resides in a duplicated inverted region on chromosome 5q. A near identical copy of the SMN1 gene, designated SMN2, contains a single nucleotide change which alters splicing, and drastically reduces the efficiency by which exon 7 is incorporated, leading to a less than full length mRNA and protein product^13^. However, SMN2 transcription produces a small percentage of full length SMN protein. An increased number of SMN2 gene copies, with the resulting increase in full-length mRNA levels and protein expression, are associated with a less severe disease phenotype in humans, and phenotypic rescue in an SMN knockout mouse model^14-18^.

The SMN protein appears to play an important role in spliceosomal small nuclear ribonucleoproteins (snRNP) biogenesis, although the precise means by which a deficiency of the SMN protein directly affects neuronal viability remains unclear. The identification of the unique mechanism by which phenotype is modified in SMA, via SMN protein expression from a near identical gene copy, has led to efforts to identify specific compounds which could shift splicing in the SMN2 message toward a higher rate of exon 7 inclusion or up-regulate SMN protein expression via an increase in SMN2 gene expression. We have recently developed an SMN protein assay in patient monocytes and platelets that appears to correlate with SMA severity, and is stable in repeated assays over up to a one year period of time.

At this time, there are no registered pharmacological treatments for SMA. However, several compounds have been identified that appear to up-regulate SMN gene expression or alter gene splicing to produce an increased ratio of full length to exon-7-deleted SMN mRNA. Some of these compounds are readily available for use in patients, and have a proven safety record in the treatment of other disorders. Two laboratories have reported preliminary data supporting the potential benefit of valproic acid (VPA) on SMN protein and mRNA levels**).** Drs. L. Brichta and B. Wirth presented data demonstrating that the level of full-length *SMN2* mRNA/ protein increased 2-4-fold in fibroblast cell lines derived from SMA patients treated with therapeutic doses (0.5-500 *µ*M) of VPA^19^. In addition, VPA increased SMN protein levels through transcription activation in organotypic hippocampal brain slices from rats. Drs. C. Sumner and K. Fischbeck reported that VPA significantly increases exon 7-containing SMN mRNA transcript and protein levels in three SMA Type 1 fibroblast cell lines after 24 hours of treatment. This increase was accentuated after 3 days of treatment^20^. VPA treatment also increased the number of gems, the intra-nuclear structures containing SMN protein. They demonstrated that VPA promoted correct splicing of the *SMN2* gene and activated the *SMN* promoter, indicating that the increase in full-length SMN levels in response to VPA may be due both to enhancement of correct splicing and to an increase of SMN2 transcription*.* Results from these studies seem to indicate that VPA and related compounds deserve further investigation as potential treatment for SMA.

We hypothesize that treatment with VPA increases expression of SMN protein via up-regulation of SMN2 gene expression, resulting in the rescue of motor neurons and allowing re-innervation. Preliminary safety data from a pilot clinical trial at the University of Utah site demonstrates that giving valproic acid without carnitine supplementation is likely to deplete carnitine in a high percentage of SMA patients over the proposed study period, potentially increasing toxicity and resulting in reduced strength and function, thus confounding results of a clinical therapeutic trial. Thus, the trial design takes this into account by examining the use of these two compounds together. A variety of outcome measures will be used to evaluate efficacy of these drugs, including: Blood Chemistries (CBC with platelets, AST, ALT, amylase, lipase, carnitine profile, basic metabolic panel, and valproic acid level); Functional Status/Strength as assessed via Modified Hammersmith Functional Motor Scale (MHFMS) and the Project Cure Functional Motor Scale, Myometry, Pulmonary Function Testing, Quantitative assessment of SMN mRNA from blood samples, Peds QL™ assessment, Max CMAP amplitude/area and Ulnar MUNE (Modified Motor Unit Number Estimation),Growth and vital sign parameters, 3-Day Dietary Record, and Dual Energy X-ray Absorptiometry (DEXA) scanning as a measure of changes in lean muscle mass over time, and investigation of genetic mechanisms which influence the severity of phenotype.

**Objectives**

The primary objective is to assess the safety, tolerability, and efficacy of a combined regimen of oral valproic acid (VPA) and carnitine in SMA patients 2-17 years of age. Secondary objectives are to refine electrophysiological and clinical techniques to help us better follow the course of patients with SMA, particularly as it relates to the primary disease process causing weakness, motor neuron dysfunction and loss. These include: 1) clinical assessments which will help accurately assess functional motor outcomes of patients, 2) electrophysiologic techniques to study and follow the course of motor neuron loss, and 3) investigation of genetic mechanisms which influence the severity of phenotype.

**Patient Selection Criteria**

SMA study patients must meet the following inclusion and exclusion criteria. Family members of eligible study participants will also be included if they choose to donate their blood for genetic testing. They will not participate in any other portion of the study.

***Inclusion Criteria***

*Cohort 1*

1. Confirmed genetic diagnosis of 5q SMA
2. SMA 2 or non-ambulatory SMA 3: SMA 2 subjects must be able to sit independently for at least 3 seconds without support
3. Age 2 to 8 years at time of enrollment

*Cohort 2*

1. Confirmed genetic diagnosis of 5q SMA
2. SMA subjects (SMA types 2 or 3) who can stand independently without braces or other support for up to 2 seconds
3. Age 3 to 17 years at time of study enrollment

***Exclusion Criteria***

*Cohort 1*

1. Modified Hammersmith Functional Motor Scale for children with SMA (MHFMS-SMA) total score < 2 or > 37
2. Need for BiPAP support > 12 hours per day
3. Spinal rod or fixation for scoliosis or anticipated need within six months of enrollment
4. Inability to meet study visit requirements or cooperate reliably with functional testing
5. Transaminases, amylase or lipase > 3.0 x normal values, WBC < 3.0 or neutropenia < 1.0, platelet count < 100 K, or hematocrit < 30 persisting over a 30 day period
6. Coexisting medical conditions that contraindicate travel, testing or study medications
7. Use of medications or supplements which interfere with valproic acid or carnitine metabolism, increase the potential risks of these medications, or are hypothesized to have a beneficial effect in SMA animal models or human neuromuscular disorders within 3 months of study enrollment. Specifically, concomitant use of riluzole, creatine, butyrate derivatives, growth hormone, anabolic steroids, daily albuterol use, anticonvulsants, or other HDAC inhibitors would preclude enrollment.
8. Current use of either VPA or carnitine through participation in another study or been prescribed by their attending physician. If study subject is taking valproic acid or carnitine then patient must be go through a washout period of 12 weeks before been enrolled into the study.
9. Body Mass Index > 90^th^ % for age

*Cohort 2*

1. Spinal rod or fixation for scoliosis or anticipated need within six months of enrollment
2. Inability to meet study visit requirements or cooperate with functional testing
3. Transaminases, amylase or lipase > 3.0 x normal values, WBC < 3.0 or neutropenia < 1.0, platelets < 100 K, or hematocrit < 30 persisting over a 30 day period.
4. Coexisting medical conditions that contraindicate travel, testing or study medications
5. Use of medications or supplements which interfere with valproic acid or carnitine metabolism, increase the potential risks of these medications, or are hypothesized to have a beneficial effect in SMA animal models or human neuromuscular disorders within 3 months of study enrollment. Specifically, concomitant use of riluzole, creatine, butyrate derivatives, growth hormone, anabolic steroids, daily albuterol use, anticonvulsants, or other HDAC inhibitors would preclude enrollment.
6. Current use of either VPA or carnitine through participation in another study or been prescribed by their attending physician. If study subjects is taking valproic acid or carnitine then patient must be go through a washout period of 12 weeks before been enrolled into the study.
7. Body Mass Index > 90^th^% for age
8. Pregnant women/girls, or those intending to try to become pregnant during the course of the study

**Design**

Implementation of multicenter phase II studies to assess safety, tolerability and efficacy of a combined regimen of oral valproic acid and carnitine will proceed in two parallel studies involving differing SMA cohorts. Preliminary safety data from a pilot clinical trial at the University of Utah site demonstrates that giving valproic acid without carnitine (C) supplementation is likely to deplete carnitine, potentially increasing toxicity and resulting in reduced strength and function, thus confounding results of a clinical therapeutic trial. Cohort 1 trial design is double-blind randomized intention to treat protocol for SMA “sitters”; parallel trial in Cohort 2 SMA “standers and walkers” to explore responsiveness of efficacy outcomes. Cohort 1 will involve SMA subjects with SMA 2 or nonambulatory SMA 3 “sitters”, 2 – 8 years of age. Cohort 2 will involve SMA 2 and 3 subjects: “standers and walkers”, 3 – 17 yrs of age.

**Study procedures**

All study patients from both cohorts will complete two baseline screening visits approximately one month apart. Upon completion of these baseline visits, patients will be divided into treatment arms and be given study medication. Patients in cohort 1 (SMA “sitters”) will be randomized to participate in either the treatment arm (group 1) or the placebo arm (group 2). Patients and investigators will remain blinded for the first six months of the study. After six months, patients in the treatment arm (group 1) will continue on study medication for an additional 6 months. All children enrolled in the placebo arm (group 2) will be switched to study medication and continue on treatment for six months. Thus, half the children will receive treatment for a full 12 months, while the other half will receive treatment for the last 6 months of the study. Patients in cohort 2 (SMA “standers and walkers”) will receive treatment for the entire 12 month time period.

Safety Labs will be drawn 2 – 4 weeks after screening visit 2 to assess safety labs and valproic acid and carnitine levels. This can be done via their local laboratories and the results would be faxed to the central study site. Any dosing adjustments needed will be coordinated through the medical monitor to keep investigators blinded until completion of the study. Additional safety labs for cohort 1 will be drawn 2-4 weeks after Screen 2, 6.5-7 months and 9 months. Additional safety labs for Cohort 2 will be drawn 2-4 weeks after Screen 2. Safety labs do not require a visit to the study site, but may be coordinated through patient’s local pediatrician.

**Cohort 1**: SMA “sitters”: 2 – 8 yrs

|  | Screen 1 | Screen 2 | Safety Lab 1 | Visit 1 | Visit 2 | Safety  Lab 2 | Safety Lab 3 | Visit 3 |
| --- | --- | --- | --- | --- | --- | --- | --- | --- |
|  | T-1 mo | T=0 | .5-1 mos | 3 mos | 6 mos | 6.5-7 mos | 9 mos | 12 mos |
| GROUP 1:  30 subjects | Baseline | Baseline  Start Drugs | VPA+ C | VPA+ C | VPA+C | VPA+C | VPA+C | VPA+C |
| GROUP 2:  30 subjects | Baseline | Baseline | Placebo x2 | Placebo x2 | Placebo x2 | VPA+C | VPA+C | VPA+C |

**Cohort 2:** SMA “standers and walkers”, ages 3 – 17 yrs

|  | Screen 1 | Screen 2 | Safety Lab 1 | Visit 1 | Visit 2 | Safety Lab 2 | Safety Lab 3 | Visit 3 |
| --- | --- | --- | --- | --- | --- | --- | --- | --- |
|  | T-1 mo | T=0 | .5-1 mos | 3 mos | 6 mos | 6.5-7 mos | 9 mos | 12 mos |
| GROUP 3:  30 subjects | Baseline | Baseline  Start Drugs | VPA+ C | VPA+ C | VPA+C | Not Neede | VPA+C | VPA+C |

Clinical testing on the first day of the study visit will include the following: a comprehensive medical history and a general physical examination including tests of blood pressure, breathing rate and oxygen level, heart rate, and measures of growth including height, weight, head circumference, chest circumference, abdominal circumference, and body fat. We estimate this will take about 20 minutes. Nerve conduction testing will be performed at each visit. This testing involves attaching electrodes (½ inch rectangular adhesive pads) over muscles in the hand. The electrodes taped over muscles in the hand will be connected to an EMG machine to record responses. Patients will lay in the position most comfortable for him/her. A nerve will be activated at the wrist and around the elbow by applying brief electrical shocks. Sedation will be necessary in some children, and will be performed under the supervision of the study site investigator, with the assistance of trained nurses to assist with the administration of medications and monitoring patient’s vital signs.  An anesthesiologist or emergency-attending physician will be immediately available should an unexpected complication ensue during the procedure.  Patients will be NPO for several hours prior to the procedure as per routine hospital sedation guidelines.  The goal is conscious sedation, to minimize anxiety and voluntary movement but not to deeply sedate the patient or even put them to sleep and its use will be carefully determined by age and ability to cooperate. Patients will be sedated using Valium 0.5-0.8 mg/kg/dose with a maximum dose of 10 mg by mouth and or Versed 0.1 – 0.3 mg/kg/dose intranasally or by mouth up to 0.5 mg/kg/dose, and may receive up to one dose every ten minutes during the procedure (maximum 2 doses).  Possible complications of the sedation could include respiratory compromise, hypotension or an unexpected paradoxical response to the medication. Heart rate, pulse oximetry, and blood pressure will be measured prior to the start of the study, monitored continuously during the study, and for 30 min following its completion or until the patient is adequately awake and has completely returned to baseline respiratory status. Suction equipment and oxygen will be available at the bedside if needed. The time required for the nerve conduction testing and monitoring is approximately 60 minutes.

Blood samples will be obtained either immediately before or after the nerve conduction testing. If the patient is going to be sedated, the blood draw will be done while the patient is still under sedation. Three to six milliliters of blood is needed at each visit to check for safety monitoring and drug levels. If patients elect to have their DNA collected for genetic studies, an additional five to ten milliliters will be collected during the course of the study. If a DEXA scan is to be completed during this visit, it will occur either immediately before or immediately after the MUNE procedure. The total time of testing on the first day, including all of the testing above, is approximately 2 hours.

Prior to the patient’s arrival for each study visit they will be given a 3 day dietary/supplement record, which they will asked to complete and bring with them. This information will be reviewed with them by a dietician either on their arrival or on the first day of testing. If patients forget to bring their completed dietary record, they will be asked to complete it the following week and return it promptly. The site coordinator will administer the parent questionnaire of the quality of life tool (PedQL^TM^), which must be filled out by the same parent at each visit. Total time to complete the questionnaires is about 15 minutes.

Clinical testing on the second day includes the following: A physical therapist will perform a functional assessment using either the Modified Hammersmith Functional Motor Scale – SMA (cohort 1) and the Project Cure Functional Motor Scale for SMA (cohort 2). Functional testing will last between 15 to 30 minutes. Patients 5 years of age or older will receive pulmonary function testing and myometry testing. A physical therapist will perform myometry testing which uses a small device to help measure strength of patients’ hand grip and arm flexion and leg extension. A technician will perform pulmonary function testing which includes routine spiromerty, maximum inspiratory pressure (MIP) and maximum expiratory pressure (MEP). This testing will take approximately 30 minutes. Patients 5 years or older will complete the age appropriate questionnaire of the quality of life tool PedQL^TM^ on this day.

Participation in this study will include 5 visits and 7 total blood tests over a 13 month period. Each visit will be two to three days long. The first day will be for travel if needed, the second day will include clinical exam, nerve conduction testing and collection of blood samples, and all functional testing will be performed on the third day. Follow-up Study Visits after beginning therapy will be at 3 months (Visit 1), 6 months (Visit 2) and 12 months (Visit 3) when the patient will stop drug. All evaluations will be performed at each follow-up visit. To account for scheduling conflicts, illnesses, or family emergencies, study visits may occur within a four week window (plus or minus 2 weeks) from the anticipated date.

The following table illustrates the study procedure outline:

|  | Screening | | Visit | | | | | |
| --- | --- | --- | --- | --- | --- | --- | --- | --- |
|  | 1 | 2 | Safety Lab 1 | 1 | 2 | Safety Lab 2 | Safety Lab 3 | 3 |
| Time - from start of drug | - 4 wks | Start | Month .5 - 1 | Month 3 | Month 6 | Month* 6.5 - 7 | Month 9 | Month 12 |
| Physical Examination and Vital signs | X | X |  | X | X |  |  | X |
| Growth Parameters | X | X |  | X | X |  |  | X |
| Blood Draw | X | X | X | X | X | X | X | X |
| Quality of Life Survey: PEDS QL^TM^  Parent version: all  Child versions: > 5 yrs | X | X |  | X | X |  |  | X |
| Nerve Conduction Testing | X | X |  | X | X |  |  | X |
| Functional Testing | X | X |  | X | X |  |  | X |
| 3 day dietary, supplements, medication survey | X | X |  | X | X |  |  | X |
| DEXA Scan** |  | X |  |  | X |  |  | X |
| Start Drug |  | X |  |  | X |  |  |  |
| Stop Drug |  |  |  |  |  |  |  | X |

*Safety Lab 2 will be done approximately at month 6.5-7, but must be scheduled to occur 2 weeks to 1 month after v2, even if V2 is not held in the window of month 6 +/- 2 weeks.

**DEXA scans apply to Cohort 1 only

VPA and placebo will be acquired from the pharmaceutical company Abbott Pharmaceutical and carnitine and placebo from Sigma-Tau Pharmaceutical. Study medications and placebos will be sent to the central pharmacy at Primary Children’s Medical Center for direct dispensing to study patients. At screening visit 2, when inclusion criteria are fulfilled, the central pharmacist will be notified to prepare a 90 day supply (+ 14 day emergency supply) and ship to study patient. At Study visit 1 (3 month follow-up), another 90 day supply will be requested by the study site investigator and distributed by the central pharmacy. Patients will receive enough extra study medication to maintain a 14 day emergency supply. At study visit 2 (6 month follow-up), a 180 day supply similarly be supplied to take the patient to Study visit 3 (12 month follow-up). At Study visit 3, study medications will be discontinued.

Dosage initiation of VPA will start at 10-20mg/kg/day divided into two or three doses. After 2 week follow-up safety lab the dose will be increased to achieve a therapeutic trough level 50-120 micrograms/ml. All subjects will receive VPA in the form of 125 mg sprinkle capsules. Dosage for Carnitor will be 50 mg/kg/day with a maximum dose of 1000 mg/day divided into two doses. Carnitor elixir comes as 500 mg/5 ml. All subjects will be given Carnitor or equivalent placebo in the liquid form. At each follow-up visit, the caregiver will be asked to present a log of drug administration and to return all unused capsules. The log and returned drug will be reviewed by the Site Coordinator and results tabulated.

While genetic confirmation of 5q SMA is necessary for inclusion in this study, that information will come from the routine clinical evaluation of patients prior to enrollment. New genetic information obtained in our study will include the SMN2 copy number, a known modifier of SMA phenotype, which will be performed in the CLIA certified laboratory of Dr. Tom Prior at Ohio State University. Blood will also be collected for mRNA studies which will be performed at Montreal in the laboratory of Dr. Louise Simard and for protein studies to be conducted at the University of Utah. We will also request consent to establish a DNA and cell line resource on each patient and their parents, so as to have this resource available to perform further genetic studies which might distinguish “responders” from “non-responders”. However, patients and parents may refuse to participate in the development of a permanent DNA and cell line database without affecting their eligibility for enrollment in this study.

**Standard of Care vs. Research-Related Procedures**: There are no standard-of-care procedures in this

study; every procedure is strictly research related. Although this study hopes to contribute to a future

standard-of-care treatment plan for SMA, as of this date no treatment has yet been approved.

**Data Safety and Monitoring:** The University of Utah General Clinical Research Center (GCRC)

Informatics Core serves as the infrastructure for a data coordinating center (DCC). The GCRC Informatics

Core is responsible for applying the best practices of data management, study design, safety reporting and

clinical trial conduct to clinical research protocols. It will also provide project direction and management,

data security and other necessary support from existing resources within the GCRC Informatics Core. There will be additional staff required for this project, a part time Database Administrator (DBA) and clinic coordinator.

The data management system is based on the Clinical Research Information System (CRIS) version 4 that has been developed, tested and implemented at the University of Utah since October 2003. Its implementation was based on a clinical study process model developed in 1999.

The DSM committee consist of 6 members who are completely independent of all investigators and have no financial, scientific or other conflict of interest with the study. A quorum is 4 voting members. The Principal Investigator and Data Management Biostatistician are non-voting members.

Colin B. Van Orman, MD Medical Monitor/Pediatric Neurologist

Thomas. F. Pajak, Ph.D Biostatistician

Robert M. Taylor, MD Ethicist

Michael Workman, PT Pediatric Physical Therapist

Nicola Longo, MD, PhD Biochemical Geneticist

Jacob V. Aranda, MD, PhD Clinical Neonatologist/Pharmacologist

Criteria for discontinuation of the study have been set by the Data Safety Monitoring Committee, in consultation with the Principal Investigator.

Patients are made aware of all potential risks during the informed consent process. Safety assessments consist of monitoring and recording all adverse events and serious adverse events and the regular monitoring of parameters including hematology, liver function tests, vital signs, and general physical condition. Blood draws will be obtained during each study visit and between some study visits to monitor safety levels.

The Medical Monitor (MM) reviews all laboratory data within a week of each visit and on an as-needed basis to determine whether any subject suffers evidence of organ damage or an adverse event and to make decisions regarding dosage adjustments. All lab results are provided on laboratory reports along with normal limits for each value. The MM notes any abnormalities and signs the case report form before returning to the Clinical Trials Manager. In addition, the MM receives monthly reports summarizing any adverse events.

**Statistical Analysis, Data Analysis and Interpretation**

*Sample Size*

In the Pilot Natural History Study conducted in the GCRC at the University of Utah, we found that SMA Type 2 patients did not significantly change over a six-month period when measured with the Modified Hammersmith Functional Motor Scale-SMA (MHFMA-SMA). The standard deviation for this longitudinal group was 3.19. The two treatment groups differ over the first six months, thus the primary outcome will be change in Modified Hammersmith Functional Motor Scale-SMA from the second baseline to six months. A clinically meaningful change is taken to be a 3-point change. A sample size of 25 patients completing both the baseline and six-month evaluations will provide 90% statistical power with an effect size of 0.941, Type I error of 0.05 (two-sided). Assuming 20% attrition, then 60 total patients will be required.

The patients that can stand or walk will be evaluated in a cohort 2 arm. The primary endpoint will be the SMA-3 scales. When the sample size is 25, a two-sided 95.0% confidence interval for a single mean will extend 39% of the standard deviation from the observed mean; the confidence interval is based on the large sample z statistic. In order to ensure that 25 patients complete the therapy then 30 patients will be accrued.

*Statistical Analyses*

The primary aim is to compare change over the first six months on the MHFMA-SMA for the VPA+C arm versus placebo. The assumption of normality of the change scores will be checked, if it holds then a t-test will be performed comparing the difference in average change scores. Gender and age may affect MHFMA-SMA scores; as such a linear regression analysis that includes gender and age will be included along with treatment. This will be a confirmatory analysis. Subjects that drop out prior to six months will be excluded from the primary analysis. If the subjects had a 3-month evaluation then an additional analysis will be performed using the last observation carried forward approach to determine the effect on the outcome of subjects with missing data.

Secondary endpoints include an evaluation of the change in MHFMA-SMA at 12 months. We will evaluate the change from second baseline to 12 months and compare both groups. This will be done using a t-test. Since all patients will cross over to VPA+C at six months, this evaluation will be exploratory to determine whether or not additional treatment time of 12 vs 6 months leads to additional functional benefit in the treatment cohort compared to the placebo cohort and compared to their baseline values. We will also look at the change in MHFMA-SMA from 6 to 12 months for both groups. In addition, generalized estimating equations will be used to incorporate all longitudinal measurements from the second baseline through 12 months to determine if there is a sustainable treatment effect. Additional secondary endpoints include: Pulmonary Function Testing in subjects over five years of age, quantitative SMN mRNA, Peds QL™, CMAP amplitude/area, and MUNE. These endpoints will be exploratory in nature. Some will be hypothesis generating, while others will be confirmatory for the MHFMA-SMA results.

*The analyses of the cohort arm will be as follows:*

The assumption of normality of the change scores will be checked, if it holds then a large-sample z-statistic confidence interval around the mean will be constructed. Change from baseline will be examined and reported with 95% confidence intervals. Gender and age may affect scores; as such a linear regression analysis that includes gender and age will be performed to determine their influence. Subjects that drop out prior to six months will be excluded from the primary analysis. If the subjects had a 3-month evaluation then an additional analysis will be performed using the last observation carried forward approach to determine the effect on the outcome of subjects with missing data.

Additional secondary endpoints include: Pulmonary Function Testing in subjects over five years of age, Quantitative assessment of SMN mRNA, Peds QL™, Maximum CMAP amplitude/area: ulnar nerve /hypothenar eminence, Ulnar MUNE, growth and vital sign parameters, dietary assessment. These endpoints will be examined at each time point with means and confidence intervals as well as an analysis over time. These exploratory analyses will aid in the design of follow-up studies in this cohort of patients.

*Randomization*

Randomization will be performed using a permuted block design balancing for institution. Each institution must submit a Study Agent Shipment Form as soon as the individual responsible for the study agent has been identified and the site has institutional IRB approval for the study. The Shipment Form must be submitted to the Central Pharmacy in order to receive drug shipment with adequate processing time before calling to register the first case. Patients can be registered only after pretreatment evaluation is completed and eligibility criteria are met. Patients are registered prior to the start of any protocol therapy by calling the Statistical Office at Children’s Hospital of Philadelphia (215) 590-3236, Monday through Friday, 8:30 a.m. to 5:00 p.m. ET. This process will be performed following screening visit 1 and verification by the lead Investigator at each study site that the subject meets all inclusion/exclusion criteria. The patient will be randomized/registered and a case number will be assigned and confirmed by email. After each randomization/registration, the Statistical Office will provide the double-blinded treatment assignment to the Central Pharmacy, who will ship the assigned therapy by next day shipment (Priority Overnight) to the institution. NOTE: Sites must plan ahead due to the following shipping schedule: Study agent/placebo for patients randomized after 4 p.m. ET will be shipped the following business day. Study agent/placebo will be shipped to U.S. sites Monday to Thursday only. Study agent/placebo will be shipped to Canadian sites Monday to Wednesday only. For example, study agent/placebo for a patient randomized to a U.S. site on Thursday after 4 p.m. will be shipped on the following Monday (or Tuesday, if Monday is a holiday). Study agent/placebo will not be shipped on holidays or December 23rd through January 2nd. Each institution is responsible for notifying the Central Pharmacy if study agent/placebo does not arrive on the expected date.

**Administrative Responsibilities**

**Study Resources:** At the University of Utah, resources include a clinic room in Primary Children’s Medical Center that is restricted to use for the research of Kathryn Swoboda and equipped with EMG equipment for MUNE and C-MAP assessments, as well as standard medical equipment (to check blood pressure, etc.); access to physical therapy and pulmonary function testing. DEXAs are performed at University Hospital; offices with computers are located in the School of Medicine; lab space is located at the SOM and at the Eccles Institute of Human Genetics, where protein studies are accomplished. We also use the GCRC for DNA extraction and send blood samples to other study sites for mRNA analysis (Montreal) and SMN copy-number (Ohio State). A Freezer maintaining -80 degrees centigrade is located in the SOM lab and houses our DNA. Qualified personnel at Utah include the PI, Clinical Trials Manager Sandra Reyna, MD; study coordinators; research dietician; research nurse; physical therapist; PFT and DEXA technicians; and informatics and nursing staff at the University of Utah General Clinical Research Center (GCRC).

**Recruitment:** We propose to recruit patients primarily from the Indiana SMA Patient Registry. When the registry receives information from an interested and eligible SMA patient, they will forward it to the study site geographically closest to the patient. Each study site will then contact the family to begin screening and enrollment procedures. This study will also advertise to interested clinicians in the journal Neurology, and directly to families on the Project Cure SMA and Families of SMA web sites. A toll free number will be established for the sole purpose of referring interested physicians and families to the nearest study site. This toll free number will be for informational purposes only and no identifying information will be collected.

**Control of Investigational Devices/Drugs:** VPA is an investigational drug insofar as it has not yet been approved for use in SMA patients. Its use is controlled by the study pharmacist at the Central Pharmacy of Primary Children’s Medical Center. She stores and dispenses VPA, Carnitine, and placeboes, mailing study drugs out under the labels of “Study Drug A” and “Study Drug B” to the parents of our pediatric subjects. Study drugs are mailed at specific times in the study to ensure proper use: after enrollment is approved, and every three months thereafter. Safety labs are required 2 weeks after start of drug/placebo and 2 weeks after all participants go on drug; safety labs are also done every 3 months, either at the time of a study visit or at a lab local to the families’ residences. The PI approves the calculation by weight of subject of the proper dosing.

**Communication Plans for Multi-Center Studies:** The investigator and coordinator(s) at each study site will be responsible for enrolling and evaluating study patients and collecting all study information. Patient samples and other patient information are given an identifying number and the code key is kept in a password protected computer. Patient files are stored according to number and are kept in locked files by the investigators. A central medical monitor will monitor laboratory values and adjust medication dosage for all sites as needed. Adverse events will be reported by investigators and coordinators at each site and will be reviewed by a data and safety monitoring board. Distribution of all medication is performed by a pharmacist at Primary Children’s Medical Center.

The GCRC Informatics Core will serve as the infrastructure for a data coordinating center (DCC). The GCRC Informatics Core will be responsible for applying the best practices of data management, study design, safety reporting and clinical trial conduct to clinical research protocols. It will also provide project direction and management, data security and other necessary support from existing resources within the GCRC Informatics Core. There will be additional staff required for this project, a part time Database Administrator (DBA) and a full time clinical coordinator. Each study center will have complete access to its own patient population date that is centrally stored at Utah. Study populations and their resulting data are combined to create a large enough sample for clinical and statistical significance. Interim results, which are already deidentified, will be compiled by the GCRC informatics director and shared with co-investigators and collaborators as the data pertains to their own specialties.

Regular conference calls including *all* multi-center staff (PIs, coordinators, data manager, physical therapists, etc.) allow for discussion of problems, possible changes to protocol, plans for interim data collection, analysis, and publication. Each conference call is summarized in minutes and distributed to all multi-center staff.

**Participating Sites outside the University of Utah:** The other five sites involved in the multi-center study are located at the Children’s Hospital of Michigan at Wayne State University; Johns Hopkins Hospital and Johns Hopkins University; University of Wisconsin Medical School, Ohio State University, and Centre de Recherche de l'Hôpital Sainte-Justine in Montreal, Canada. Each of the site PIs are named co-investigators of this study. All participating sites maintain IRB approval and are equipped to handle safety concerns as they may arise. Safety of information is handled as described above: participants’ private health information is deidentifed before being sent to the central data site at Utah and then kept in locked cabinets. Participants’ health safety is protected in numerous ways: reactions to the study drug through regular lab draws, oversight of labs by the Central Lab coordinator, an unblinded study coordinator, and the Medical Monitor; reporting of Adverse Events as stipulated by the IRBs and medical follow-up as needed; and overnight care at the different university’s GCRCs or equivalent.

**References and Appendices**

1. Brahe C, Bertini E. Spinal muscular atrophies: recent insights and impact on molecular diagnosis. J Mol Med. 1996;74:555-562

2. Roberts DF, Chavez J, Court SD. The genetic component in child mortality. Arch Dis Child. 1970;45:33-38

3. Pearn J. Incidence, prevalence, and gene frequency studies of chronic childhood spinal muscular atrophy. J Med Genet. 1978;15:409-413

4. Czeizel A, Hamula J. A hungarian study on Werdnig-Hoffmann disease. J Med Genet. 1989;26:761-763

5. Emery AE. Population frequencies of inherited neuromuscular diseases--a world survey. Neuromuscul Disord. 1991;1:19-29

6. Merlini L, Stagni SB, Marri E, Granata C. Epidemiology of neuromuscular disorders in the under-20 population in Bologna Province, Italy. Neuromuscul Disord. 1992;2:197-200

7. Pearn J. Classification of spinal muscular atrophies. Lancet. 1980;1:919-922

8. Bromberg MB, Swoboda KJ. Motor unit number estimation in infants and children with spinal muscular atrophy. Muscle Nerve. 2002;25:445-447

9. Swoboda K, Prior T, Scott C et al. Natural History of Denervation in SMA: Relation to Age, SMN2 copy and Function. Annals of Neurology. 2005;Accepted for Publication

10. Crawford TO. From enigmatic to problematic: the new molecular genetics of childhood spinal muscular atrophy. Neurology. 1996;46:335-340

11. Gilliam TC, Brzustowicz LM, Castilla LH et al. Genetic homogeneity between acute and chronic forms of spinal muscular atrophy. Nature. 1990;345:823-825

12. Melki J, Lefebvre S, Burglen L et al. De novo and inherited deletions of the 5q13 region in spinal muscular atrophies. Science. 1994;264:1474-1477

13. Monani UR, Lorson CL, Parsons DW et al. A single nucleotide difference that alters splicing patterns distinguishes the SMA gene SMN1 from the copy gene SMN2. Hum Mol Genet. 1999;8:1177-1183

14. Campbell L, Potter A, Ignatius J et al. Genomic variation and gene conversion in spinal muscular atrophy: implications for disease process and clinical phenotype. Am J Hum Genet. 1997;61:40-50

15. Lefebvre S, Burlet P, Liu Q et al. Correlation between severity and SMN protein level in spinal muscular atrophy. Nat Genet. 1997;16:265-269

16. Monani UR, Sendtner M, Coovert DD et al. The human centromeric survival motor neuron gene (SMN2) rescues embryonic lethality in Smn(-/-) mice and results in a mouse with spinal muscular atrophy. Hum Mol Genet. 2000;9:333-339

17. Feldkotter M, Schwarzer V, Wirth R et al. Quantitative analyses of SMN1 and SMN2 based on real-time lightCycler PCR: fast and highly reliable carrier testing and prediction of severity of spinal muscular atrophy. Am J Hum Genet. 2002;70:358-368

18. Mailman MD, Heinz JW, Papp AC et al. Molecular analysis of spinal muscular atrophy and modification of the phenotype by SMN2. Genet Med. 2002;4:20-26

19. Brichta L, Hofmann Y, Hahnen E et al. Valproic acid increases the SMN2 protein level: a well-known drug as a potential therapy for spinal muscular atrophy. Hum Mol Genet. 2003;12:2481-2489

20. Sumner CJ, Huynh TN, Markowitz JA et al. Valproic acid increases SMN levels in spinal muscular atrophy patient cells. Ann Neurol. 2003;54:647-654

# 
